# The ITGB2‐COPS3‐SOX2 Axis and SOX2 Liquid‐Liquid Phase Separation: Dual Mechanisms Governing Osteosarcoma Stemness

**DOI:** 10.1002/advs.202520913

**Published:** 2026-03-13

**Authors:** Lei Guo, Zhiqing Zhao, Wei Wang, Jianfang Niu, Bing Wang, Yunfei Lin, Wei Guo, Taiqiang Yan

**Affiliations:** ^1^ Department of Orthopedics Peking University First Hospital Peking University Beijing China; ^2^ Musculoskeletal Tumor Center Peking University People's Hospital Peking University Beijing China; ^3^ Beijing Key Laboratory of Musculoskeletal Tumor Beijing China

**Keywords:** cancer stem cell, COPS3, liquid‐liquid phase separation, osteosarcoma, small‐molecule inhibitor

## Abstract

Osteosarcoma is a highly malignant bone tumor prevalent in adolescents, whose therapeutic challenges stem largely from cancer stem cell (CSC)‐mediated chemoresistance and pulmonary metastasis. High‐frequency amplification of the 17p11.2 chromosomal region in osteosarcoma represents a key molecular event driving malignant progression. This study focuses on the key gene COPS3 in this region, delving into its molecular mechanisms regulating cancer stemness. The findings show that elevated COPS3 expression strongly correlates with stemness features. Mechanistically, COPS3 binds directly to transcription factor SOX2, inhibiting its ubiquitin‐mediated degradation, enhancing its stability and transcriptional activity, and establishing a positive feedback loop that reinforces the stemness phenotype. Furthermore, the extracellular matrix receptor ITGB2 emerges as an upstream regulator promoting COPS3 nuclear translocation. Significantly, this study provides the first evidence that SOX2 undergoes liquid‐liquid phase separation (LLPS) in osteosarcoma, a critical mechanism for maintaining cancer stemness. Based on these findings, we identified Z‐5891, a highly selective COPS3 inhibitor that suppresses tumor growth and reduces cancer stemness in vitro and in vivo. This study delineates a complete stemness regulatory pathway from microenvironmental signals to nuclear LLPS, thereby providing a novel theoretical framework and a candidate drug for targeting CSCs to overcome drug resistance and metastasis in osteosarcoma.

## Graphical abstract

Osteosarcoma stemness is driven by the ITGB2‐COPS3‐SOX2 signaling axis. This study reveals that nuclear COPS3 stabilizes SOX2, which in turn undergoes liquid‐liquid phase separation to promote stemness. Based on this mechanism, a novel COPS3 inhibitor, Z‐5891, was developed, effectively suppressing tumor growth and stemness in vivo, offering a promising therapeutic strategy.

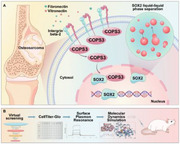

## 1 Introduction

Osteosarcoma is a highly malignant bone tumor originating from mesenchymal tissue, primarily occurring in children and adolescents [1, 2]. This tumor is characterized by high invasiveness, rapid progression, and a high tendency to metastasize to the lungs, with lung metastasis being the primary cause of patient mortality [3, 4]. Over the past few decades, the introduction of neoadjuvant chemotherapy and advances in surgical techniques have significantly improved patient prognosis, raising the five‐year survival rate to 50%–60% [5, 6, 7]. However, survival rates have not shown further significant improvement over the past 40 years, indicating that lung metastases and chemotherapy resistance remain major challenges in current clinical practice. Notably, many patients still experience metastasis during or after treatment, suggesting that existing therapeutic approaches struggle to effectively eliminate populations of tumor cells that possess drug resistance and high metastatic potential. Consequently, developing novel therapeutic strategies targeting such cells to improve patient prognosis has become an urgent priority in osteosarcoma research.

Increasing evidence indicates that cancer stem cells (CSCs) play a central role in chemotherapy resistance, recurrence, and metastasis in osteosarcoma [8, 9, 10]. CSCs constitute a cell subpopulation with self‐renewal and multipotent differentiation capabilities. They maintain tumor heterogeneity through symmetric or asymmetric division, drive disease progression, and mediate chemotherapy resistance or distant metastasis [11]. Consequently, targeting CSCs holds promise as a potential therapeutic strategy to improve the prognosis of osteosarcoma patients. Recently, liquid‐liquid phase separation (LLPS) has garnered significant attention as a crucial mechanism for the organisation of biomacromolecules. By facilitating the formation of membrane‐less organelles, it participates in regulating multiple key biological processes, including gene transcription, chromatin dynamics, and signal transduction [12]. Furthermore, it plays a pivotal role in the initiation and progression of various malignant tumours [13]. It is noteworthy that the role of LLPS in maintaining the stemness of CSCs has gradually been elucidated, suggesting its potential involvement in the core regulatory network of CSCs [14, 15, 16]. Consequently, identifying key factors specific to osteosarcoma that sustain stemness and thoroughly analysing their regulatory mechanisms not only aids in revealing novel molecular mechanisms underlying disease progression but also offers potential therapeutic strategies for overcoming drug resistance and inhibiting metastasis.

The genome of osteosarcoma is highly complex. Beyond the limited high‐frequency mutations conventionally identified, such as those in TP53 and RB1, no coding gene mutations with clear driver significance have been discovered to date. Current research generally holds that the genomic mutations in osteosarcoma differ significantly from those in other common solid tumours. The osteosarcoma genome is characterised by frequent copy number amplification and structural variations [5]. The overexpression of oncogenes resulting from these copy number variations is considered a key molecular event in the initiation and maintenance of osteosarcoma. Among these, the chromosome 17p11.2 region stands as one of the most frequently amplified zones in osteosarcoma [17]. This region harbours both genuine driver mutation genes and a substantial number of passenger genes. Therefore, identifying its core pathogenic components and elucidating their functional mechanisms holds significant importance for understanding the pathogenesis of osteosarcoma. COPS3 is precisely located within the 17p11.2 region. Previous research from our team was the first to demonstrate that COPS3 amplification constitutes a significant cause of genomic instability in this region and is closely associated with lung metastasis in osteosarcoma [18]. Subsequent independent studies, including our own work, have consistently reported COPS3 amplification occurring in a proportion of osteosarcoma [19, 20, 21]. This suggests that targeting COPS3 may hold clear clinical translational value and potentially benefit a substantial patient population. More importantly, our team's latest research has discovered that COPS3 not only promotes lung metastasis but also significantly enhances chemotherapy resistance [22, 23]. These two are precisely the most representative malignant phenotypes of cancer stem cells. Therefore, we hypothesise that COPS3 may play a pivotal role in the malignant progression of osteosarcoma by regulating the stemness maintenance mechanisms of CSCs. Against this backdrop, elucidating the molecular mechanisms underlying COPS3‐mediated stemness regulation will not only provide novel insights into the biology of osteosarcoma stem cells but also offer a crucial theoretical foundation for developing targeted therapeutic strategies and improving patient outcomes.

**SCHEME 1:**
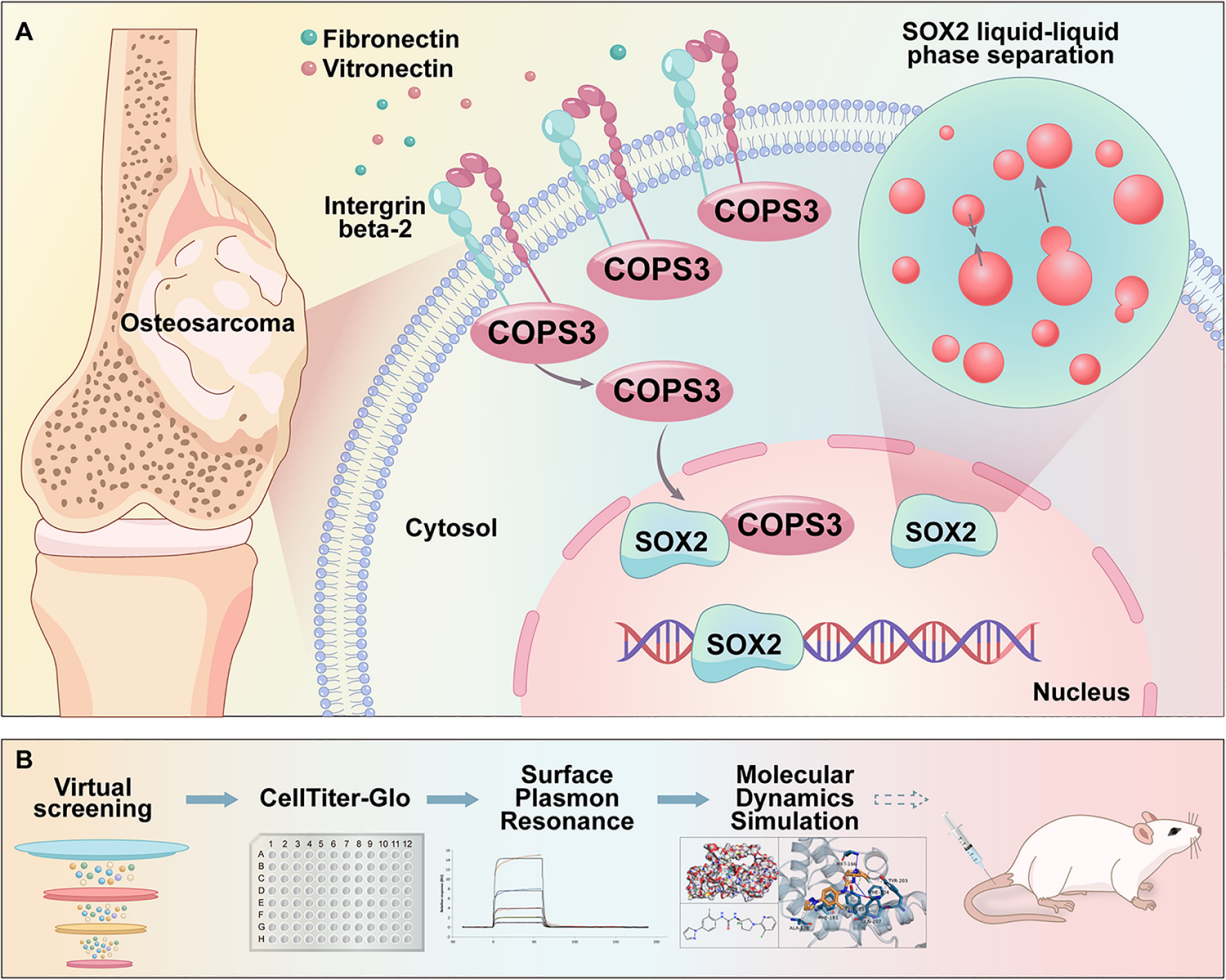
Dual mechanisms of cancer stemness regulation in osteosarcoma and targeted therapeutic strategies. (A) Dual mechanisms of ITGB2‐COPS3‐SOX2 axis and SOX2 liquid‐liquid phase separation in regulating cancer stemness in osteosarcoma. (B) Screening process for small‐molecule inhibitors targeting COPS3.

In summary, this study aimed to systematically elucidate the function of COPS3 in maintaining stemness in osteosarcoma cells and its underlying molecular mechanisms. By integrating single‐cell RNA sequencing analysis, in vitro functional assays, and clinical sample validation, this study found that COPS3 overexpression is significantly correlated with cancer stemness characteristics. The study further demonstrated that COPS3 binds to the transcription factor SOX2 protein, inhibiting its ubiquitination and degradation, thereby enhancing its protein stability and transcriptional activity, and forming a positive feedback loop that further reinforces the stem‐like phenotype of osteosarcoma cells. Meanwhile, this study identified the extracellular matrix receptor ITGB2 as an upstream regulatory molecule that enhances the functionality of the COPS3‐SOX2 signalling axis by promoting COPS3 nuclear translocation. Of particular significance, we discovered that SOX2 can form biomolecular aggregates via a liquid‐liquid phase separation mechanism and demonstrated its crucial role in maintaining osteosarcoma stemness. Based on the aforementioned mechanism, we successfully screened a highly selective small‐molecule inhibitor targeting COPS3, which significantly suppressed tumour growth and inhibited cancer stemness in both in vitro and in vivo models. This study revealed a cascade regulatory mechanism linking the ITGB2‐COPS3‐SOX2 signalling axis to the liquid‐liquid phase separation function of SOX2, providing novel insights into osteosarcoma stem cell biology and identifying a potential new therapeutic target and candidate drug for the targeted treatment of osteosarcoma (Scheme 1).

## 2 Results

### 2.1 High Expression of COPS3 Maintains Stemness in Osteosarcoma Cells

**FIGURE 1:**
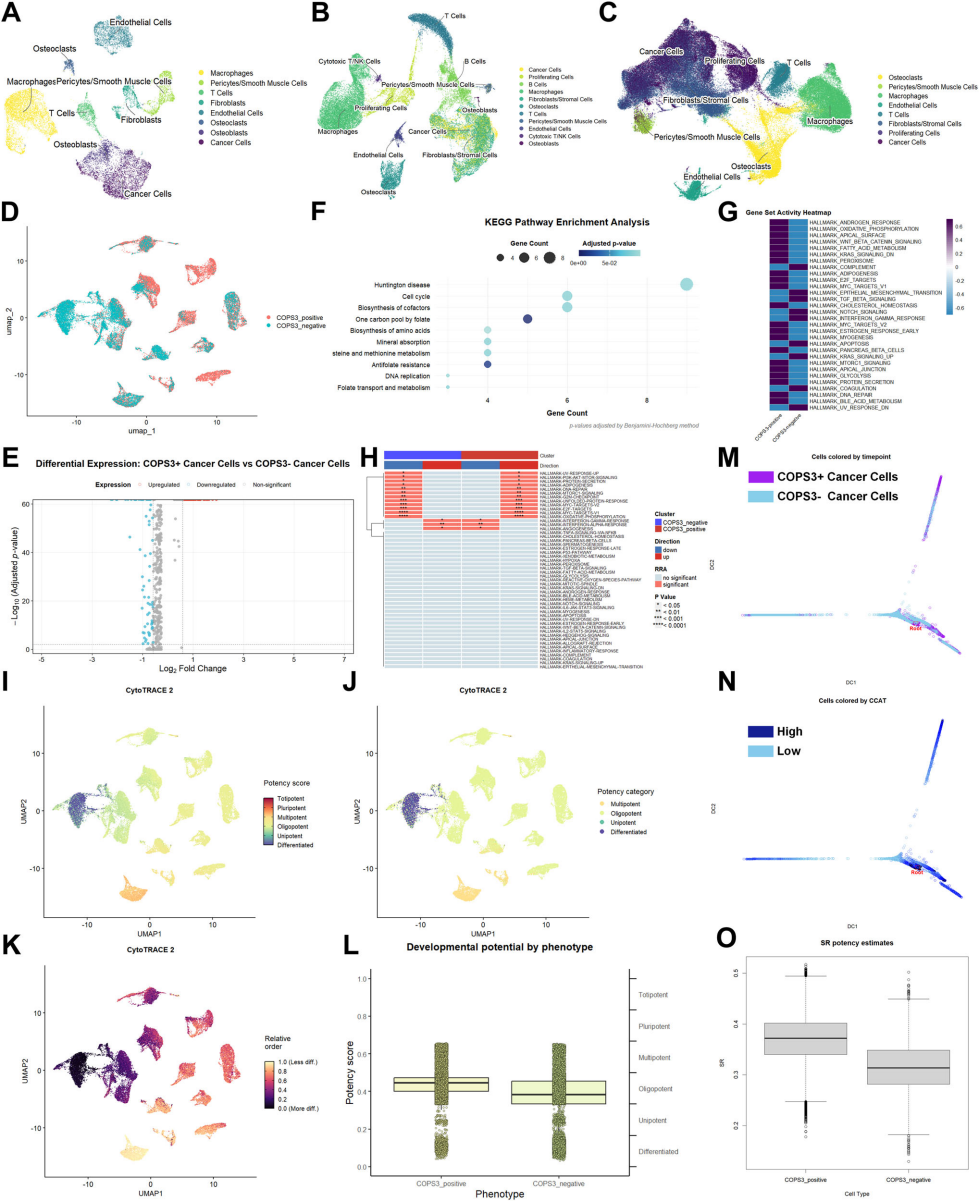
Single‐cell RNA sequencing results reveal that COPS3 maintains osteosarcoma cell stemness. (A) GSE250015 integrated dimension reduction UMAP plot. (B) GSE162454 integrated dimension reduction UMAP plot. (C) GSE152048 integrated dimension reduction UMAP plot. (D) Dimension reduction plot of single‐cell data from cancer cells. (E) Volcano plot for differential analysis of single‐cell data in cancer cells. (F) COPS3+ cancer cell KEGG enrichment plot. (G) COPS3+ cancer cells GSVA enrichment plot. (H) COPS3+ cancer cells irGSEA enrichment plot. (I) Cytotrace2 potency score dimension reduction plot. (J) Cytotrace2 potency category dimension reduction plot. (K) Cytotrace2 relative stemness dimension reduction plot. (L) Comparison of differentiation levels between COPS3+ cancer cells and COPS3‐ cancer cells. (M) Differentiation trajectory plot of COPS3+ cancer cells and COPS3‐ cancer cells. (N) CCAT score trajectory plot. (O) Stemness score of COPS3+ cancer cells and COPS3‐ cancer cells.

The study utilised the public single‐cell RNA sequencing datasets GSE152048, GSE162454, and GSE250015, employing integrated analysis via Seurat and Harmony to identify cellular subpopulations within the osteosarcoma microenvironment (Figure 1A–C; Figure S1A–F). Following the identification of tumour cells via CNV calculation, we classified these cells into two groups based on COPS3 expression levels namely COPS3+ and COPS3‐ (Figure 1D; Figure S2A–D). Differential expression analysis revealed 188 genes significantly overexpressed in COPS3^+^ cells (Figure 1E). KEGG and GSEA enrichment analyses indicated these genes primarily involved processes such as the cell cycle and DNA replication (Figure 1F; Figure S2E, Supporting Information). Further GSVA and irGSEA analyses demonstrated significant activation of CSC‐related pathways including WNT and MYC signalling pathways in COPS3^+^ cells (Figure 1G,H). Subsequently, all four scoring algorithms AUCell, UCell, singscore and ssGSEA validated that the aforementioned pathways exhibited heightened activity in COPS3^+^ cells (Figure S3A–L). Stemness assessment revealed that the COPS3^+^ cell cluster exhibited higher stemness scores and greater differentiation potential in both Cytotrace2 and SCENT analyses (Figure 1I–O; Figure S3M–P). These findings suggested that COPS3 may represent a potential regulatory factor in maintaining osteosarcoma cell stemness.

**FIGURE 2:**
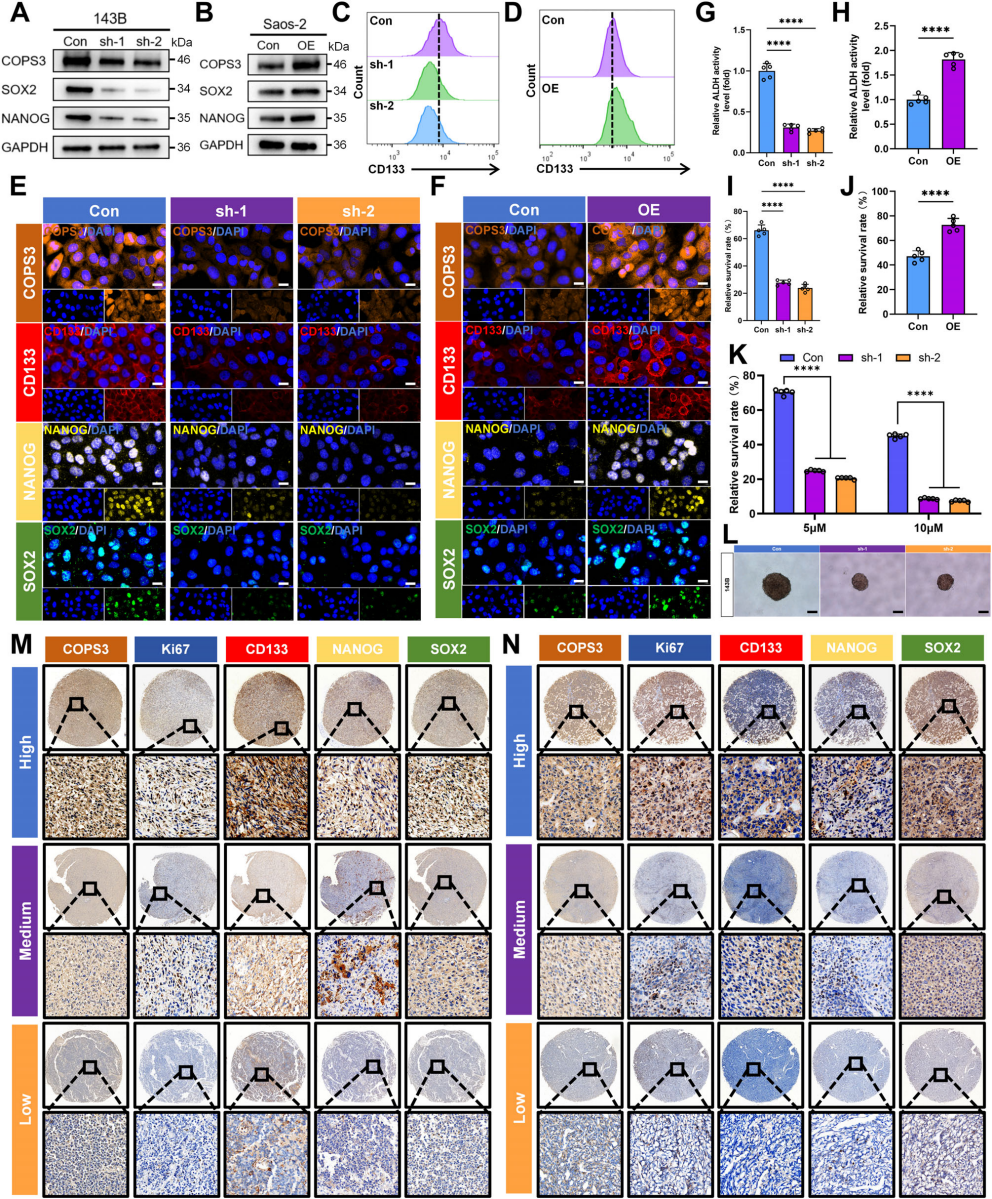
High expression of COPS3 maintains stemness in osteosarcoma cells. (A‐B) Effects of COPS3 knockdown (A) or overexpression (B) on the expression of CSC‐related markers in cells were detected by Western blotting. (C‐D) Effects of COPS3 knockdown (C) or overexpression (D) on CD133 expression in cells were detected by flow cytometry. (E‐F) Effects of COPS3 knockdown (E) or overexpression (F) on the expression of CSC‐related markers in cells were detected by immunofluorescence staining (scale bar, 20 µm). (G‐H) Effect of COPS3 knockdown (G) or overexpression (H) on ALDH activity in cells, n = 5. (I‐J) Effect of COPS3 knockdown (I) or overexpression (J) on the ability of cells to resist anoikis, n = 5. (K) Effect of knockdown of COPS3 on the ability of 143B cells to resist doxorubicin, n = 5. (L) Effects of knockdown of COPS3 on the ability of cells to sphere formation (scale bar, 200 µm). (M) Expression of CSC‐related markers in osteosarcoma primary tissues (scale bar, 500and 20 µm respectively). (N) Expression of CSC‐related markers in osteosarcoma lung metastatic tissues (scale bar, 500 and 20 µm respectively). Results are reported as means ± SD. **** p‐value <0.0001.

We first determined the endogenous protein levels of COPS3 across osteosarcoma cell lines. Western blot (WB) analysis revealed that 143B cells expressed the highest level of COPS3, followed by KHOS‐240S cells, while Saos‐2 cells expressed the lowest level (Figure S4A). Based on this expression profile, we selected 143B cells for COPS3 knockdown experiments and Saos‐2 cells for COPS3 overexpression experiments to investigate its role in regulating CSC properties. Western blot analysis revealed that COPS3 knockdown significantly reduced the protein expression levels of multiple CSC‐associated markers, whereas COPS3 overexpression markedly upregulated the expression of these markers (Figure 2A,B). Flow cytometry analysis further confirmed that COPS3 knockdown resulted in a marked decrease in the proportion of CD133‐positive cells, whereas its overexpression increased the proportion of CD133‐positive cells (Figure 2C,D; Figure S4B,C). Immunofluorescence (IF) staining similarly demonstrated a positive correlation between COPS3 expression levels and the expression of CSC markers (Figure 2E,F; Figure S4D–K). Furthermore, this study evaluated acetaldehyde dehydrogenase (ALDH) activity in relation to CSC function. The results demonstrated that COPS3 knockdown markedly reduced cellular ALDH activity, whereas COPS3 overexpression significantly enhanced this activity (Figure 2G,H). CSCs typically exhibit resistance to anoikis, a characteristic closely associated with tumor metastasis. By culturing cells in ultra‐low attachment 96‐well plates to simulate anoikis conditions, we found that COPS3 knockdown significantly impaired cell viability under suspension, whereas COPS3 overexpression markedly enhanced the capacity of cells to resist anoikis (Figure 2I–J). Chemotherapy resistance is another key characteristic of CSCs. Following 48 h of doxorubicin treatment, COPS3‐knockdown cells exhibited increased sensitivity to doxorubicin, whereas the overexpressing cells demonstrated enhanced resistance (Figure 2K; Figure S4L). To precisely quantify the impact of COPS3 on the self‐renewal capacity of osteosarcoma stem cells, we performed limiting dilution assays followed by Extreme Limiting Dilution Analysis (ELDA). In the 143B cell line, knockdown of COPS3 profoundly diminished the frequency of sphere‐initiating cells. The estimated stem cell frequency was 1 in 10.1 for the control group, whereas it dropped significantly to 1 in 63.0 for the COPS3‐sh1 group and 1 in 85.2 for the COPS3‐sh2 group (Figure S4M). Consistent with this quantitative assessment, representative images confirmed a marked reduction in the size of clonally derived spheres upon COPS3 knockdown (Figure 2L). In contrast, overexpression of COPS3 significantly enhanced the self‐renewal capacity of Saos‐2 cells. The stem cell frequency increased from 1 in 44.32 in the control group to 1 in 8.02 in the COPS3‐overexpressing group (Figure S4N,O). Finally, immunohistochemical (IHC) analysis was conducted on primary osteosarcoma tissue from 42 patients and lung metastatic tissue from 28 patients. The results demonstrated a significant positive correlation between COPS3 expression and the expression of multiple CSC markers, thereby validating the aforementioned conclusions in clinical samples (Figure 2M,N; Figure S5A–H).

### 2.2 COPS3 Maintains Osteosarcoma Stemness by Stabilizing SOX2 and Inhibiting its Ubiquitin‐Mediated Degradation

**FIGURE 3:**
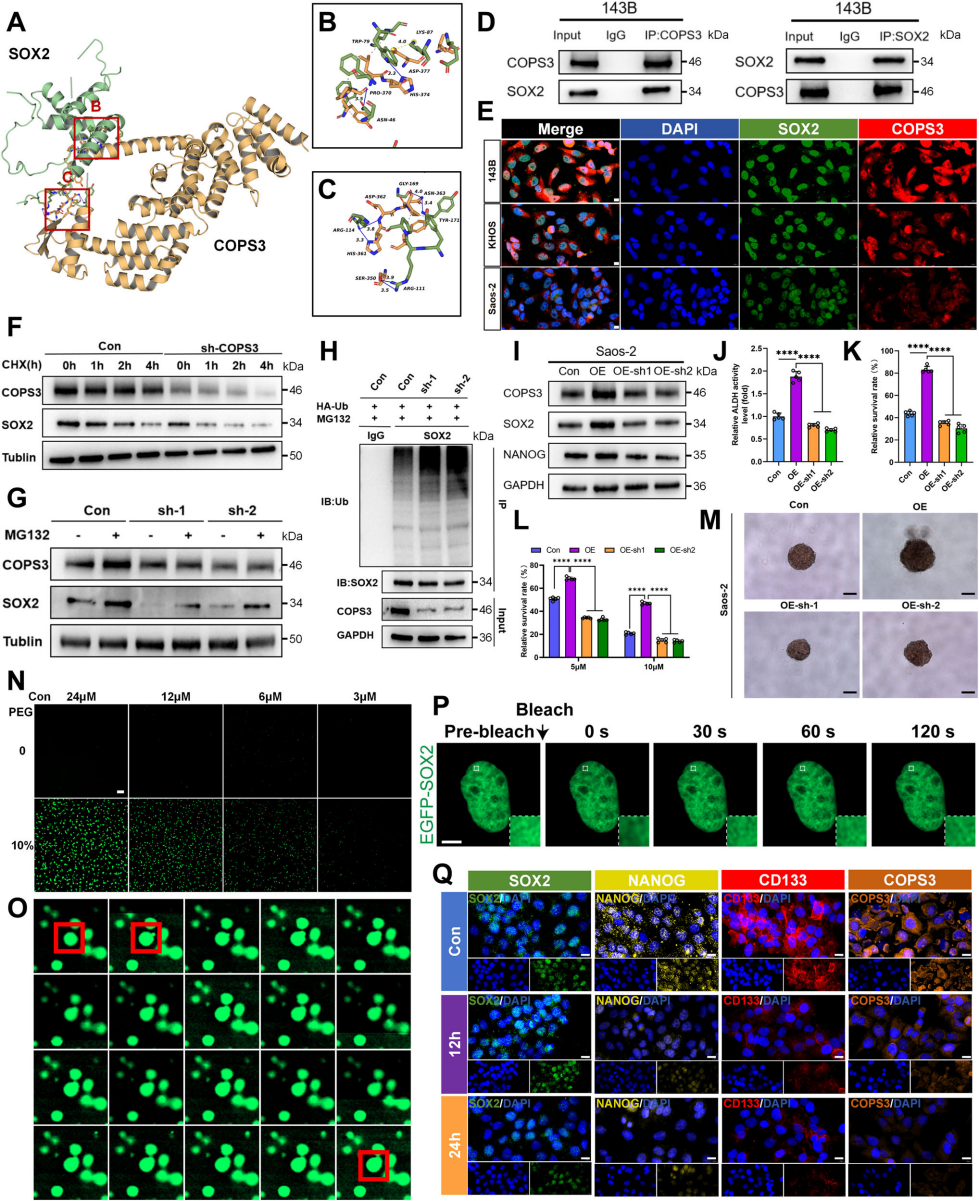
COPS3 stabilizes SOX2 by inhibiting its ubiquitination and promotes cancer stemness properties in osteosarcoma. (A–C) Interaction between COPS3 (yellow) and SOX2 (green) analyzed by PLIP. (D) Co‐immunoprecipitation verified COPS3 binding to SOX2. (E) Immunofluorescence staining verified the co‐localization of COPS3 with SOX2 (scale bar, 10 µm). (F) 143B cells with and without knockdown of COPS3 were subjected to CHX (50 µg/ml) assay. (G) Effect of the proteasome degradation inhibitor MG132 on SOX2 protein expression levels in COPS3 knockdown or non‐knockdown 143B cells. (H) The effect of COPS3 on SOX2 ubiquitination was analyzed after treatment with MG132 (20 µM) for 6 h. (I) COPS3 affected the expression of CSC‐related markers in Saos‐2 cells by affecting SOX2. (J) COPS3 affected ALDH activity in Saos‐2 cells by affecting SOX2, n = 5. (K) COPS3 affected the ability of Saos‐2 cells to resist anoikis by affecting SOX2, n = 5. (L) COPS3 affected the ability of Saos‐2 cells to resist doxorubicin by affecting SOX2, n = 5. (M) COPS3 affected the ability of Saos‐2 cells to sphere formation by affecting SOX2 (scale bar, 200 µm). (N) In vitro imaging of the SOX2 protein (scale bar, 10 µm). (O) Representative time‐lapse images showing the fission (split) and fusion (merge) events of SOX2 protein droplets in vitro. Red boxes highlight these dynamic processes, demonstrating the liquid‐like properties of the condensates. (P) Intracellular FRAP (scale bar, 5 µm). (Q) Immunofluorescence staining of CSC‐related markers in 143B cells after treatment with 1,6‐hexanediol (scale bar, 20 µm). ****p < 0.0001.

Having established the role of COPS3 in maintaining osteosarcoma cell stemness, we further investigated its potential molecular mechanisms. As a member of the high‐mobility group (HMG) transcription factor family, SOX2 has been extensively reported to play a pivotal role in CSCs. Given the above studies demonstrating that COPS3 influences SOX2 expression levels, we hypothesise that COPS3 may maintain cellular stemness by regulating SOX2. This study utilised the protein interaction prediction platform Genemania to analyse and identify a specific binding interaction between COPS3 and SOX2 (Figure S6A). Subsequently, molecular docking analysis was conducted using the full‐length AlphaFold‐predicted structure of SOX2 (UniProt ID: P48431) as the receptor and COPS3 (UniProt ID: Q9UNS2) as the ligand. The HDOCK results indicated that the optimal model exhibited a binding energy of ‐241.34 with a confidence level of 0.8614, demonstrating substantial binding potential between the two molecules (Figure S6B). The surface model clearly delineated the structural features of the binding interface between COPS3 and SOX2 (Figure S6C). The binding free energy calculated using the MM/GBSA method was ‐41.14 kcal/mol, further supporting the existence of a stable interaction between COPS3 and SOX2. Concurrently, this study identified multiple hydrogen bonds and hydrophobic interactions between COPS3 and SOX2, including a salt bridge between SOX2 LYS‐87 and COPS3 ASP‐377, and a hydrogen bond between TRP‐79 and HIS‐374 (Figure 3A–C). Subsequent co‐immunoprecipitation (Co‐IP) confirmed their interaction (Figure 3D), whilst IF analysis showed co‐localization of COPS3 and SOX2 in 143B, KHOS, and Saos‐2 cells (Figure 3E; Figure S6D–F).

In the aforementioned experiments, we observed that knocking down COPS3 resulted in a significant reduction in SOX2 protein levels while mRNA levels remained unchanged, suggesting that COPS3 may influence the stability of SOX2 (Figure S6G,H). Treatment of 143B cells with CHX revealed that COPS3 knockdown markedly shortened the half‐life of SOX2 (Figure 3F). Furthermore, treatment with the proteasome inhibitor MG132 reversed the degradation of SOX2 induced by COPS3 knockdown and partially restored SOX2 expression in cells (Figure 3G). Subsequent investigations revealed significantly elevated levels of SOX2 ubiquitination in COPS3 knockdown cells, suggesting that COPS3 may maintain SOX2 stability by inhibiting ubiquitin‐proteasome pathway‐mediated protein degradation (Figure 3H).

To determine whether SOX2 mediates the regulatory effect of COPS3 on stemness, we overexpressed COPS3 in Saos‐2 cells and observed a significant enhancement of CSC‐related phenotypes. These changes were accompanied by increased expression of stemness markers, elevated ALDH activity, enhanced resistance to anoikis, improved doxorubicin resistance, and augmented tumorsphere formation capacity as quantified by limiting dilution analysis. In contrast, knockdown of SOX2 under the COPS3‐overexpressing background reversed the aforementioned phenotypes, indicating that SOX2 serves as a critical downstream effector through which COPS3 regulates stemness properties (Figure 3I–M; Figure S6I). Furthermore, chromatin immunoprecipitation (ChIP)‐PCR assays confirmed that SOX2 directly binds to the promoters of COPS3, NANOG, and CD133, thereby activating their transcription, which demonstrates the ability of SOX2 to transcriptionally regulate multiple stemness‐associated genes (Figure S6J–L).

### 2.3 SOX2 Drives Osteosarcoma Stemness Properties Through Liquid‐Liquid Phase Separation

Notably, the SOX2 protein contains an intrinsically disordered region, suggesting its potential to undergo LLPS [24, 25]. Our study demonstrated that purified SOX2 forms concentration‐dependent droplets in vitro, exhibiting classic phase separation behavior that could be inhibited by 1,6‐hexanediol (Figure 3N,O; Figure S7A–E). Moreover, intracellular fluorescence recovery after photobleaching (FRAP) assays revealed that SOX2 condensates exhibit dynamic fluidity, consistent with the characteristics of LLPS (Figure 3P; Figure S7F). To further investigate the role of SOX2 phase separation in its function, we treated 143B cells with 1,6‐hexanediol. We found that although SOX2 protein levels remained largely unaltered, the protein expression of its downstream stemness‐related genes was significantly reduced, demonstrating that LLPS inhibition disrupts the transcriptional activation function of SOX2 (Figure 3Q; Figure S7G–J). Similarly, LLPS inhibition markedly attenuated ALDH activity, resistance to anoikis, and tumorsphere formation capacity as assessed by limiting dilution analysis, demonstrating that SOX2‐mediated LLPS is crucial for maintaining CSC properties (Figure S7K–N).

### 2.4 ITGB2 Regulates Stemness in Osteosarcoma Cells by Affecting COPS3

**FIGURE 4:**
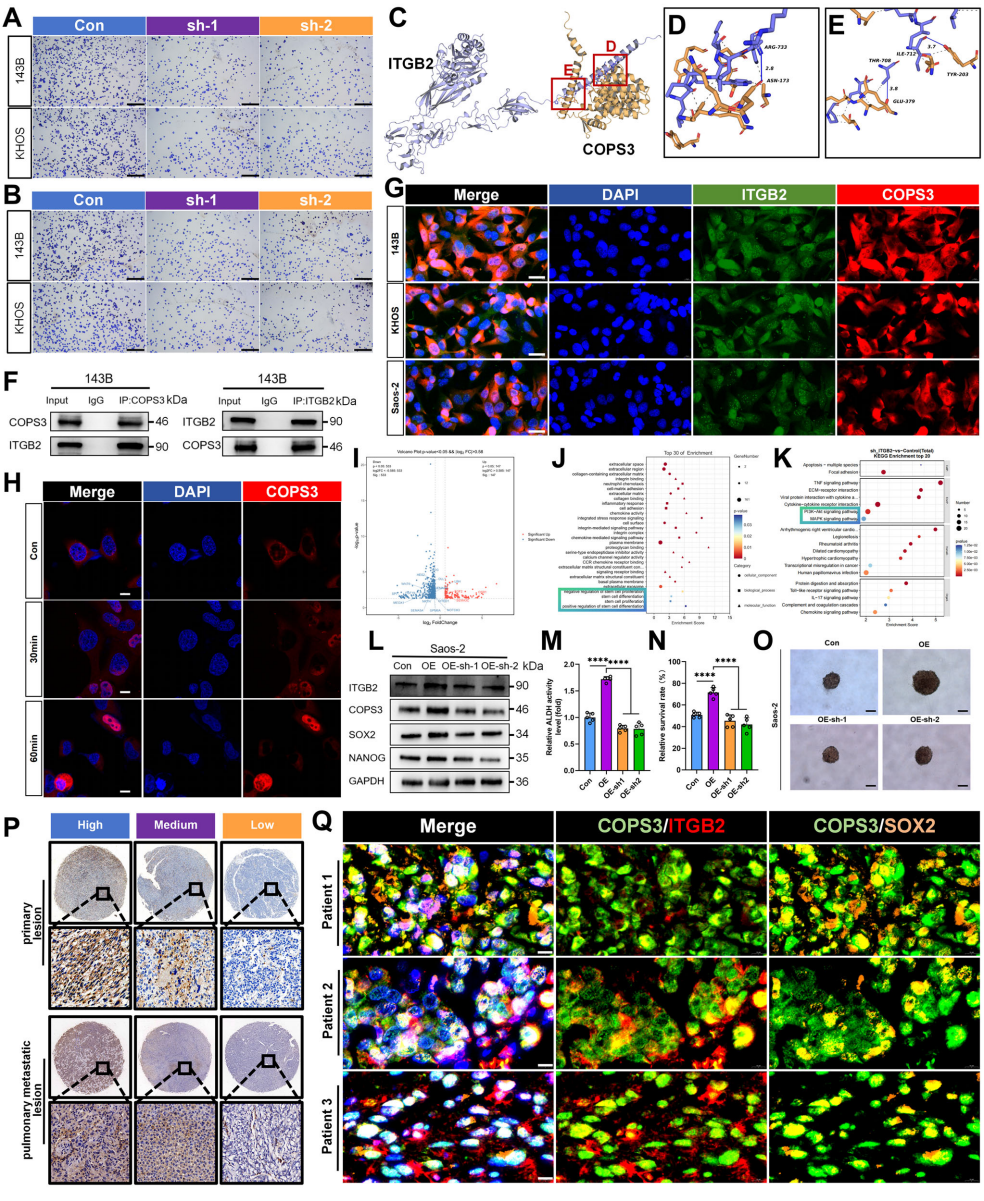
ITGB2 regulates stemness in osteosarcoma cells by affecting COPS3. (A‐B) Effect of knockdown of COPS3 on the ability of 143B and KHOS cells to adhere to vitronectin (A) and fibronectin (B). (C–E) Interaction between COPS3 (yellow) and ITGB2 (purple) analyzed by PLIP. (F) Co‐immunoprecipitation verified COPS3 binding to ITGB2. (G) Immunofluorescence staining verified the co‐localization of COPS3 with ITGB2 (scale bar, 20 µm). (H) Confocal microscopy showing that stimulation with the ITGB2 agonist Leukadherin‐1 (5 µM) promoted COPS3 nuclear accumulation in 143B cells (scale bar, 10 µm). (I) DEGs obtained by RNA sequencing between 143B cells with knockdown of ITGB2 and 143B cells without knockdown of ITGB2. (J) GO enrichment analysis of DEGs. (K) KEGG analysis of DEGs. (L) ITGB2 affected the expression of CSC‐related markers in Saos‐2 cells by affecting COPS3. (M) ITGB2 affected ALDH activity in Saos‐2 cells by affecting COPS3, n = 5. (N) ITGB2 affected the ability of Saos‐2 cells to resist anoikis by affecting COPS3, n = 5. (O) ITGB2 affected the ability of Saos‐2 cells to sphere formation by affecting COPS3 (scale bar, 200 µm). (P) ITGB2 expression in osteosarcoma primary tissues and osteosarcoma lung metastatic tissues (scale bar, 500 and 20 µm respectively). (Q) Colocalization analysis of COPS3 and ITGB2, as well as COPS3 and SOX2, in osteosarcoma primary tissues (scale bar, 10 µm). COPS3: green; ITGB2: red; SOX2: orange. Results are reported as means ± SD. ****p < 0.0001.

Although the mechanism of COPS3 in regulating cancer stemness through SOX2 has been preliminarily established, its upstream regulatory signals remain unclear. Previous studies have demonstrated that interactions between CSCs and the extracellular matrix (ECM) can significantly influence their stemness properties [26]. In the aforementioned experiments, we observed that knockdown of COPS3 markedly impaired cellular adhesion to the ECM (Figures 4A,B; Figure S8A,B). Given that integrins serve as key receptors mediating cell‐matrix interactions and potentially bridge extracellular signals with intracellular pathways, we hypothesize that COPS3 may interact with specific integrin family members to facilitate the transduction of extracellular signals into the cell, thereby regulating osteosarcoma stemness. To test this hypothesis, we first constructed a protein‐protein interaction network for COPS3 and utilized the Genemania database, which predicted a potential specific interaction between COPS3 and the integrin subunit ITGB2 (Figure S8C). We further evaluated the binding affinity between these two proteins through molecular docking. The AlphaFold‐predicted structures of ITGB2 (Uniprot: P05107) and COPS3 (Uniprot: Q9UNS2) were employed as the receptor and ligand respectively, with docking performed using HDOCK. Results indicated that the model with the lowest binding energy achieved a docking score of ‐260.86 with a confidence level of 0.9018 (Figure S8D), suggesting strong binding potential between the two. This conclusion was further supported by the surface representation (Figure S8E) and the MM/GBSA‐calculated binding free energy of ‐78.31 kcal/mol. Systematic analysis of the binding interface using PLIP and pyMOL revealed that three hydrogen bonds (distance < 4.1 Å) could form between ITGB2 and COPS3. These interactions involve COPS3 (ASN‐173) with ITGB2 (ARG‐733), COPS3 (TYR‐203) with ITGB2 (ILE‐712), and COPS3 (GLU‐379) with ITGB2 (THR‐708) (Figure 4C–E). The interaction between ITGB2 and COPS3 was further validated by Co‐IP assays (Figure 4F). Consistent with this finding, IF staining revealed distinct co‐localization of both proteins in 143B, KHOS, and Saos‐2 cell lines (Figure 4G; Figure S8F–H).

Notably, following activation of ITGB2 signaling, we observed a significant increase in COPS3 accumulation within the nucleus, suggesting that ITGB2 may regulate COPS3 function by promoting its nuclear translocation (Figure 4H). To further investigate the role of ITGB2 in stemness regulation, we performed RNA sequencing on 143B cells with or without ITGB2 knockdown. The results showed that ITGB2 depletion significantly altered the expression of multiple stemness‐associated genes (Figure 4I; Table S1). GO and KEGG enrichment analyses further indicated that several pathways associated with the maintenance of cancer stemness were significantly affected (Figure 4J,K).

Based on these findings, we hypothesized that ITGB2 regulates osteosarcoma stemness through COPS3. We established stable ITGB2‐overexpressing Saos‐2 cells and further knocked down COPS3 in this context. The results demonstrated that ITGB2 overexpression significantly enhanced the expression of stemness‐related markers, whereas COPS3 knockdown effectively reversed this effect (Figure 4L). Similarly, in functional assays assessing ALDH activity, anoikis resistance and doxorubicin resistance, COPS3 knockdown markedly attenuated the ITGB2 overexpression‐enhanced stemness phenotypes (Figure 4M,N; Figure S8I). Tumorsphere formation assays as determined by limiting dilution analysis consistently supported this finding (Figure 4O; Figure S8J). Finally, IHC analysis of 42 primary and 28 pulmonary metastatic osteosarcoma specimens demonstrated a significant positive correlation between ITGB2 expression and multiple CSC markers, thus providing clinical validation for the proposed mechanism (Figure 4P; Figure S9A–J). Moreover, IF analysis of clinical osteosarcoma specimens revealed significant co‐localization of ITGB2 with COPS3, as well as COPS3 with SOX2, thereby not only confirming the existence of the ITGB2‐COPS3‐SOX2 signaling axis in patient tissues but also providing critical evidence for its clinical relevance as a functional pathway in osteosarcoma (Figure 4Q; Figure S9K–P).

### 2.5 Integrated Virtual Screening and Molecular Dynamics Simulations Identify Z‐5891 as a Potent COPS3‐Targeted Lead Compound Against Osteosarcoma

Based on these findings, we have elucidated a signaling axis that transmits extracellular cues into the nucleus, demonstrating its critical role in maintaining osteosarcoma stemness and establishing COPS3 as a potential therapeutic target for osteosarcoma. Building upon this discovery, we are committed to developing small‐molecule inhibitors targeting COPS3 protein function to disrupt this signaling axis, thereby suppressing osteosarcoma proliferation and stem cell properties.

**FIGURE 5:**
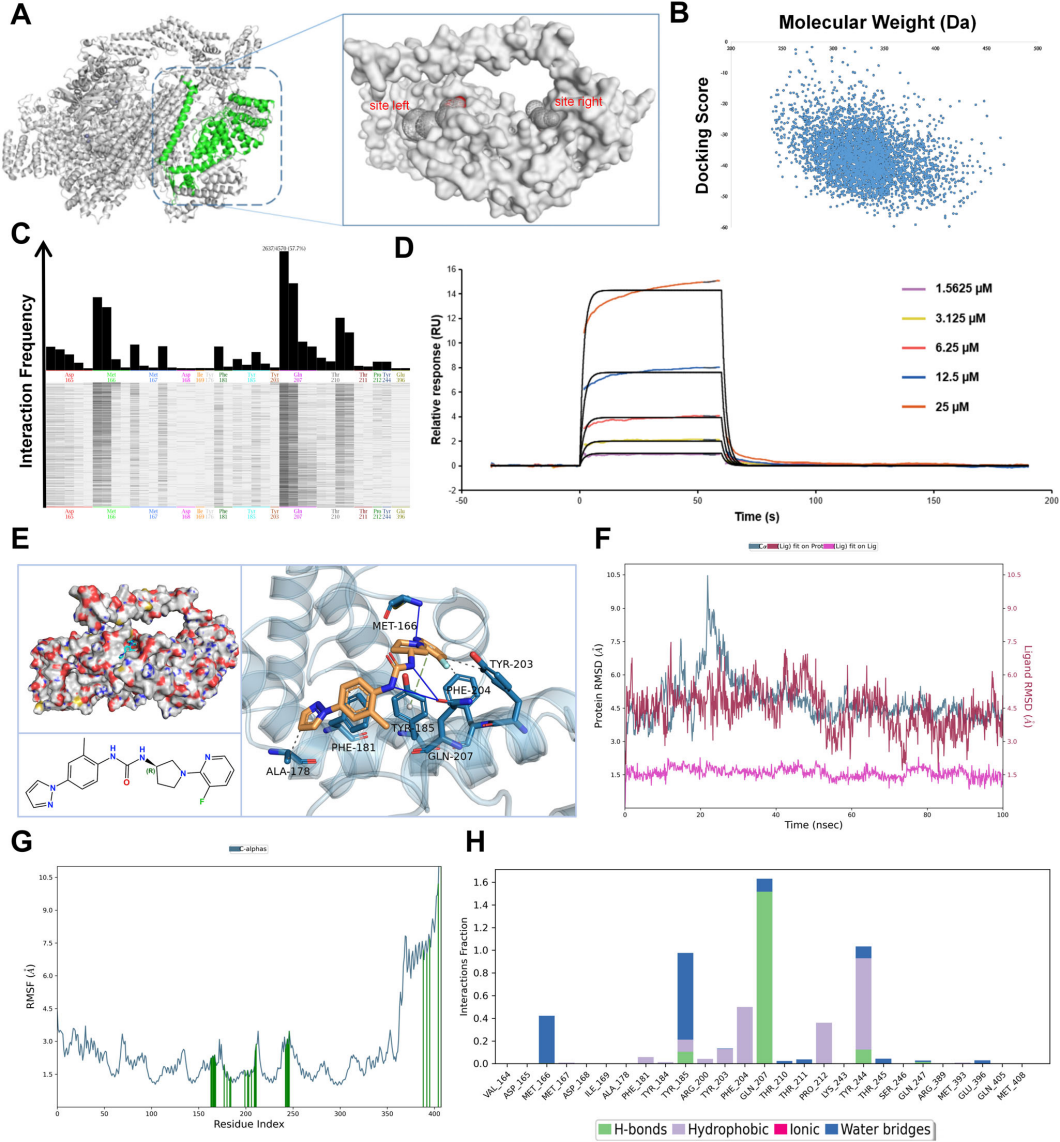
Screening and molecular dynamics simulation of small‐molecule inhibitors for COPS3 protein. (A) MOE performed pocket prediction for protein COPS3, with the site‐left and site‐right pockets identified as having higher confidence levels. (B) Relationship between scoring results and molecular weight in the database. (C) COPS3 protein and screening results PLIF analysis, where identical amino acids are represented by the same color. Higher vertical density indicates a greater frequency of interaction with the compound. (D) Detection of the interaction between Z‐5891 and COPS3 proteins via SPR. (E) 3D interaction model diagram of COPS3 protein and Z‐5891. (F) RMSD analysis. (G) RMSF analysis. (H) Interaction frequency between COPS3 protein and Z‐5891.

In this study, we first performed structural preprocessing of the COPS3 protein (PDB ID: 8H38) and predicted its binding pockets using MOE software. Based on the prediction scoring results, the site‐left pocket with high confidence (score: 4.26; threshold >3.0) was selected as the target for virtual screening (Figure 5A). The Enamine Hit Locator Library (containing approximately 460,000 small molecules) was preprocessed and used as the ligand library for molecular docking. This screening process initially generated 5,085 conformations, with 4,626 unique molecules retained after deduplication. The scoring results obtained from the database are shown in Figure 5B. There is no significant linear correlation between these scores and molecular weight, indicating that the screening outcomes were not dominated by molecular weight. This reflects the validity of the scoring function and the rationality of the screening strategy. Subsequent protein‐ligand interaction fingerprint (PLIF) analysis revealed that the majority of molecules exhibited significant interactions with amino acid residues within the binding pocket, with MET166, GLN207, and THR210 demonstrating particularly high interaction frequencies (Figure 5C). After excluding 56 molecules lacking detectable interactions, 4,570 compounds were retained for subsequent analysis. From these results, 130 molecules with MM/GBSA binding free energies below −50 kcal/mol were selected for structural diversity clustering analysis. A structural similarity threshold of 70% was applied, yielding 109 clusters. The molecule with the optimal score was retained from each cluster, resulting in the selection of 109 molecules. Subsequently, the ADMET Lab 3.0 platform was employed to assess the drug‐like properties and PAINS characteristics of these molecules. After excluding compounds that violated the Lipinski, Pfizer, or GSK rules, along with those exhibiting binding energies above −50 kcal/mol, 52 small molecules were ultimately selected for experimental validation.

This study employed the CellTiter‐Glo (CTG) assay to evaluate the inhibitory effects of these 52 compounds on the proliferation of osteosarcoma 143B cells, identifying 15 compounds capable of significantly suppressing cell growth. Subsequent surface plasmon resonance (SPR) analysis was employed to evaluate their binding affinity to the COPS3 protein, leading to the identification of a lead small molecule, Z‐5891 (Figure 5D; Figure S10A), which exhibited high binding affinity and achieved over 90% inhibition rate in 143B cells. Subsequently, molecular docking and molecular dynamics simulations were performed to characterize the interaction between Z‐5891 and COPS3. The docking results indicated that the Pose1 conformation showed the most favorable binding energy score (‐6.47 kcal/mol), forming hydrophobic interactions with ALA178, PHE181, TYR203, and PHE204, hydrogen bonds with MET166 and GLN207, and a π–π stacking interaction with TYR185 (Figure 5E; Tables S2 and S3). Subsequently, molecular dynamics simulations were conducted with Pose1 from molecular docking as the initial structure. RMSD analysis indicated that the complex system reached equilibrium after approximately 30 ns, with ligand fluctuations remaining below 3Å throughout the process, demonstrating excellent binding stability (Figure 5F). RMSF results indicated that the PCI domain within the COPS3 protein, spanning residues 1‐365, exhibited minimal fluctuations (<4.5Å), whereas the disordered region beyond residue 365 showed significant motion, consistent with known structural characteristics (Figure 5G). Further analysis of the specific amino acid interactions between Z‐5891 and COPS3 revealed that MET166, TYR185, PHE204, GLN207, and TYR244 in COPS3 were key residues for maintaining binding, exhibiting the highest interaction frequency. The interactions involved hydrogen bonding, hydrophobic contacts, and water bridge formations (Figure 5H). The trajectory from 80–100 ns (801‐1000 frames) was selected for MM/GBSA binding free energy calculation, yielding an average value of −60.25 kcal/mol. These results collectively indicate that the Z‐5891‐COPS3 complex exhibits extensive interactions, reflecting a strong and stable binding between the two molecules.

### 2.6 Targeting COPS3 With Z‐5891 Suppresses Malignant Phenotypes and Stemness in Osteosarcoma

**FIGURE 6:**
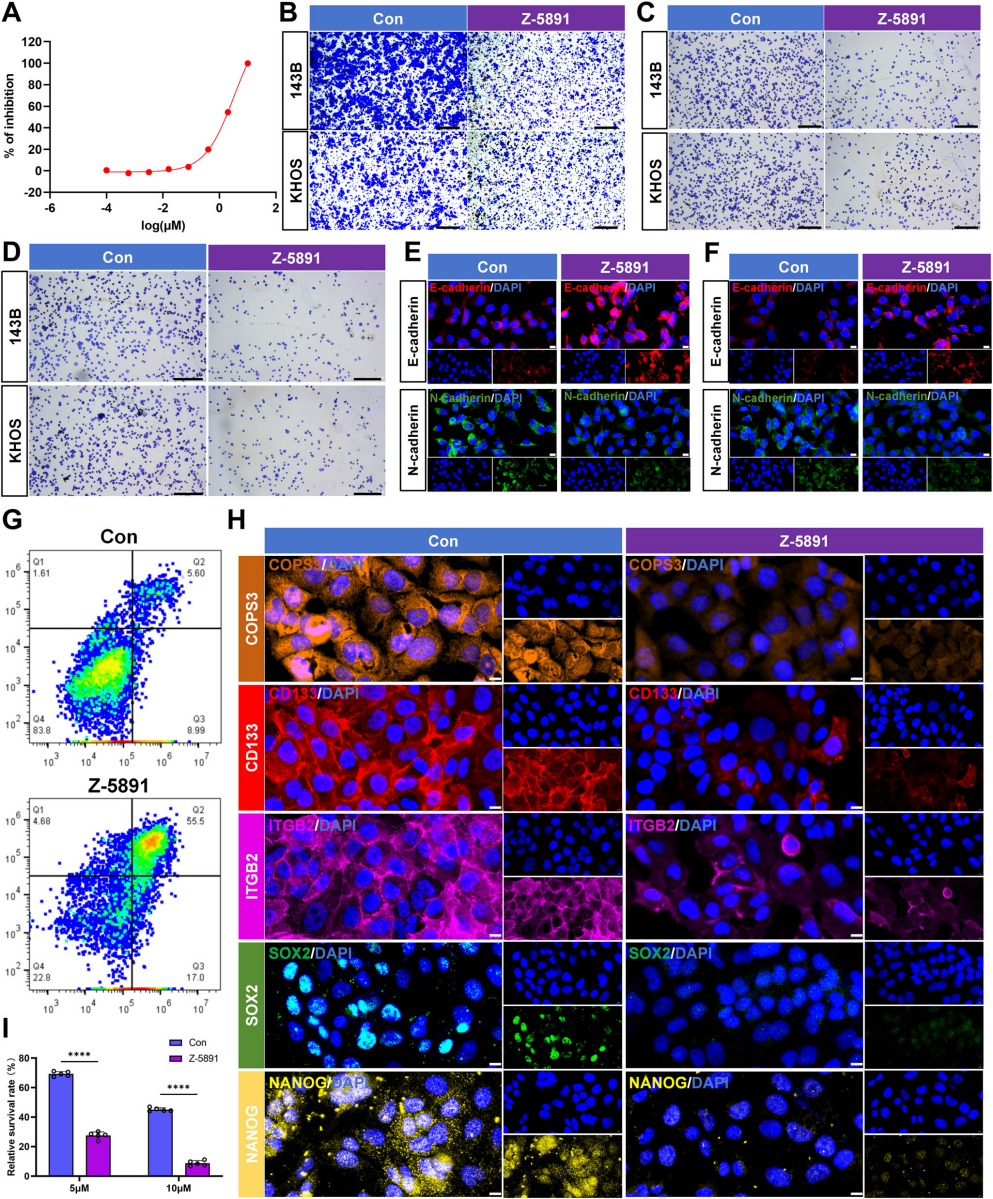
Z‐5891 suppresses multiple malignant phenotypes in osteosarcoma cells. (A) Z‐5891 exhibits concentration‐dependent proliferation‐inhibitory activity against 143B cells. (B) Effect of Z‐5891 treatment (3.28 µM for 143B, 5.06 µM for KHOS) on the invasive ability in 143B and KHOS cells. (C‐D) Effect of Z‐5891 treatment (3.28 µM for 143B, 5.06 µM for KHOS) on the ability of 143B and KHOS cells to adhere to vitronectin (C) and fibronectin (D). (E‐F) Immunofluorescence detection of the effect of Z‐5891 treatment (3.28 µM for 143B, 5.06 µM for KHOS) on E‐cadherin and N‐cadherin expression in 143B (E) and KHOS (F) cells. (G) Flow cytometric analysis of apoptosis in 143B cells treated with Z‐5891 (3.28 µM). (H) Immunofluorescence staining of CSC‐related markers in 143B cells after treatment with Z‐5891 (3.28 µM) (scale bar, 10 µm). (I) Effect of Z‐5891 treatment (3.28 µM) on the ability of 143B cells to resist doxorubicin, n = 5. Results are reported as means ± SD. ****p < 0.0001.

To further evaluate the therapeutic potential of the small‐molecule inhibitor Z‐5891, this study systematically assessed its capacity to suppress malignant phenotypes such as tumour proliferation and inhibit cancer stemness at the cellular level. We determined the IC_50_ of Z‐5891 in three osteosarcoma cell lines with varying endogenous COPS3 expression levels. The IC_50_ was 3.28 µM in 143B cells (Figure 6A), 5.06 µM in KHOS cells, and 13.41 µM in Saos‐2 cells (Figure S10B,C). The trend of higher COPS3 expression being associated with greater sensitivity to Z‐5891 (lower IC_50_) further supports COPS3 as the functional target of this compound. Transwell assay results demonstrated that Z‐5891 treatment significantly inhibited cell migration and invasion capabilities (Figure 6B; Figure S11A). Furthermore, the adhesion capacity of osteosarcoma cells to the ECM was significantly reduced following Z‐5891 treatment (Figure 6C,D; Figure S11B,C). IF staining revealed that the expression of the epithelial marker protein E‐cadherin was significantly upregulated in Z‐5891‐treated cells, while the expression of the mesenchymal marker protein N‐cadherin was simultaneously downregulated, indicating that the epithelial‐mesenchymal transition (EMT) process was effectively inhibited (Figure 6E,F; Figure S11D,E). Apoptosis analysis via flow cytometry revealed a markedly increased apoptotic rate in Z‐5891‐treated cells, confirming the pro‐apoptotic effect of targeting COPS3 (Figure 6G; Figure S11F). This study subsequently investigated the impact of Z‐5891 on cancer stemness. Results revealed a marked reduction in the expression of key cancer stemness‐associated markers (Figure 6H; Figure S11G–M). Consistent with this, Z‐5891 treatment substantially diminished ALDH activity, anoikis resistance, doxorubicin resistance and tumorsphere formation capacity as determined by limiting dilution analysis (Figure 6I; Figure S11N–Q). Collectively, these findings demonstrate that targeting COPS3 effectively suppresses cancer stemness characteristics in osteosarcoma cells.

### 2.7 The COPS3 Inhibitor Z‐5891 Synergizes With Doxorubicin to Suppress Osteosarcoma Growth and Reverse Cancer Stemness

**FIGURE 7:**
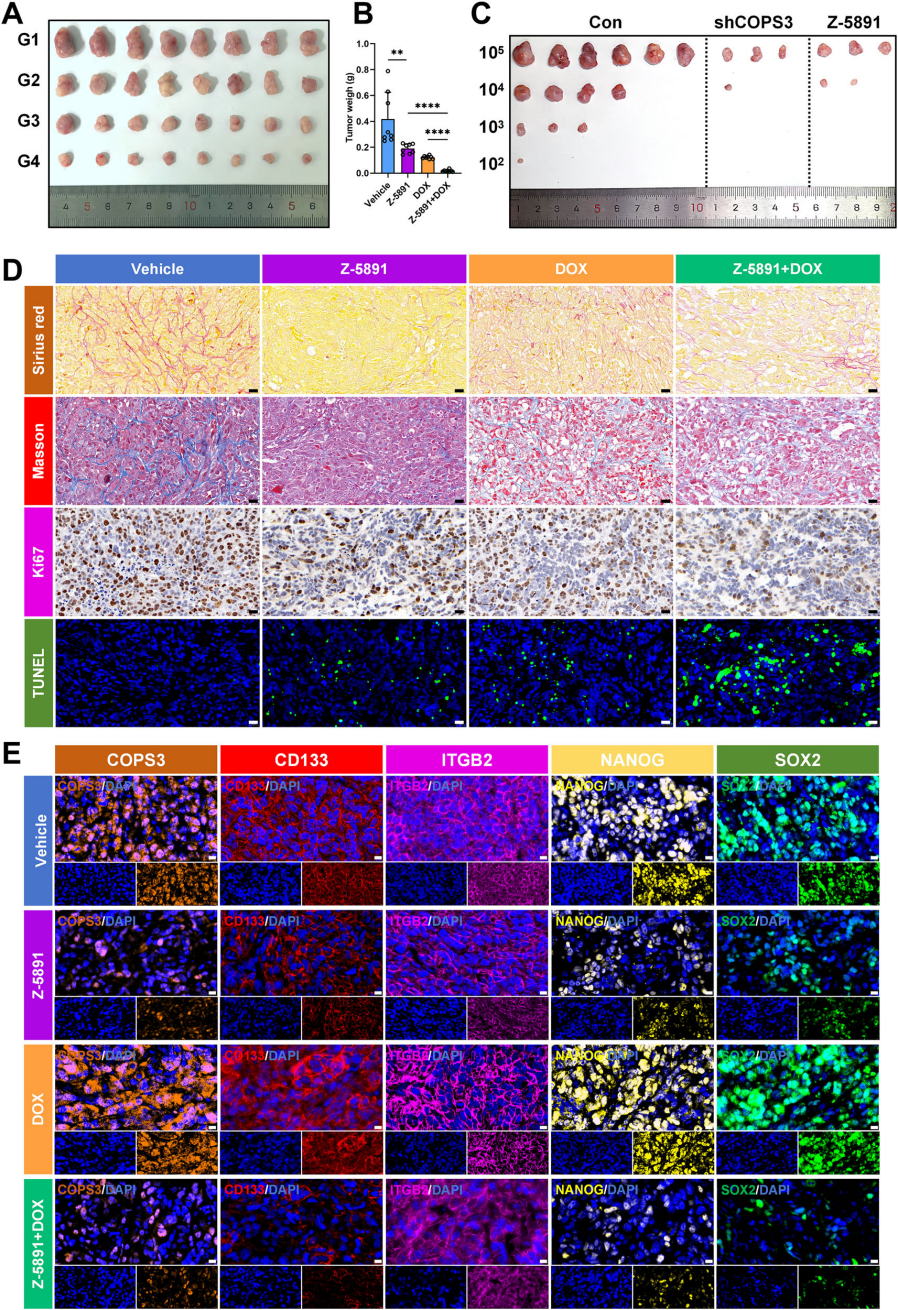
Z‐5891 synergizes with doxorubicin to suppress osteosarcoma tumor growth and cancer stemness in a mouse xenograft model. (A) The combination of Z‐5891 and DOX demonstrated potent antitumor activity in a mouse subcutaneous osteosarcoma xenograft model. G1: vehicle group; G2: Z‐5891 treatment group; G3: DOX treatment group; G4: Z‐5891 and DOX treatment group. (B) Quantitative analysis of tumor weights in each group. (C) Representative images of tumor‐bearing mice from Control, shCOPS3, and Z‐5891 groups six weeks post‐inoculation. (D) Sirius red, masson's trichrome, Ki67 and TUNEL staining results for each group (scale bar, 20 µm). (E) Immunofluorescence staining of CSC‐related markers results for each group (scale bar, 10 µm). **p < 0.01, ****p < 0.0001.

To evaluate the antitumour efficacy of COPS3 small‐molecule inhibitors in vivo, we established a subcutaneous osteosarcoma mouse xenograft model. Mice were then randomly assigned to the vehicle group (G1), Z‐5891 treatment group (G2), DOX treatment group (G3), and combined Z‐5891 and DOX treatment group (G4). As shown in Figure 7A, compared with the vehicle group, both Z‐5891 monotherapy and DOX monotherapy significantly inhibited tumour growth, manifested as a markedly slowed tumour volume growth curve and a significant reduction in tumour mass at the experimental endpoint (Figures 7A,B; Figure S12A). Notably, the combination therapy group of Z‐5891 and DOX exhibited the strongest antitumour effect, with both tumour volume and mass significantly lower than either monotherapy group, suggesting a synergistic interaction between the two agents (Figure 7B; Figure S12A). To definitively evaluate the effect of COPS3 on the tumor‐initiating capacity of osteosarcoma stem cells in vivo, we performed a limiting dilution tumor initiation assay. Control, shCOPS3, and Z‐5891‐pretreated 143B cells were inoculated into immunocompromised mice at doses of 100,000, 10,000, 1,000, and 100 cells (six mice per dose per group). Extreme limiting dilution analysis revealed that COPS3 suppression profoundly reduced the frequency of tumor‐initiating cells. The frequency was 1 in 4,816 for the Control group, compared to 1 in 122,851 for the shCOPS3 group and 1 in 96,178 for the Z‐5891 group (Figure 7C; Figure S12B). These results demonstrate that both genetic and pharmacological inhibition of COPS3 severely impairs the in vivo tumor‐propagating capacity of osteosarcoma cells. To further investigate the potential mechanisms of the small‐molecule inhibitor, IHC analysis was performed on tumour tissues. Sirius red staining and masson's trichrome staining revealed significantly reduced collagen fibre deposition in all treatment groups compared to the vehicle group, indicating suppressed tumour matrix remodelling and suggesting Z‐5891 may influence tumour microenvironment architecture (Figure 7D). Ki67 staining revealed a significant reduction in the proliferation index across all treatment groups, indicating that Z‐5891 effectively diminished tumour cell proliferation capacity (Figure 7D). Concurrently, TUNEL assay results demonstrated that the apoptosis rates in all treatment groups were significantly higher than those in the vehicle group, with the highest number of apoptotic cells observed in the combination therapy group (Figure 7D). It indicated that Z‐5891 may exert its antitumour effects by inducing apoptosis. Of particular importance, we assessed the cancer stemness of each group. IF staining for classic CSC markers including CD133, NANOG, and SOX2 revealed that Z‐5891 monotherapy significantly reduced expression of these markers (Figure 7E; Figure S12C–G). In stark contrast, DOX monotherapy not only failed to diminish but actually increased the expression of CSC‐associated markers to some extent. However, when Z‐5891 was combined with DOX, this DOX‐induced phenomenon was completely reversed, and a significant reduction in cancer stemness was similarly achieved.

To evaluate the biosafety of the COPS3 small‐molecule inhibitor Z‐5891, this study conducted histological analysis of major organs and serum biochemical testing. HE staining revealed no significant pathological alterations in the heart, liver, spleen, lungs, or kidneys (Figure S12H). Concurrently, serum ALT and AST levels showed no statistically significant differences between the control and treatment groups, remaining within normal ranges (Figure S12I‐J). These findings indicated that Z‐5891, administered at the effective dose, did not induce detectable organ damage or hepatic dysfunction, demonstrating favourable safety profiles.

## 3 Discussion

Tumour heterogeneity represents one of the core characteristics of malignant tumours, driven by complex mechanisms that are intrinsically linked to tumour initiation, progression, and therapeutic resistance. In recent years, numerous studies have demonstrated that CSCs serve as a key source of heterogeneity in various solid tumors, including osteosarcoma, not only driving recurrence and metastasis but also acting as a major contributor to chemotherapy resistance [27, 28]. Given that CSCs exhibit significant genetic and phenotypic diversity across different tumor types, identifying distinct CSC markers specific to osteosarcoma and elucidating their regulatory mechanisms are of paramount importance for developing targeted therapeutic strategies against this cell population, thereby offering new research avenues to overcome current treatment limitations and improve patient prognosis.

This study systematically elucidated the pivotal role of COPS3 in regulating osteosarcoma stemness and unraveled a hierarchical regulatory cascade progressing from the ITGB2‐COPS3‐SOX2 signaling axis to SOX2 LLPS that governs stemness properties in osteosarcoma. Through multiple functional assays and clinical specimen analyses, this study validated that COPS3 promotes stemness in osteosarcoma cells by enhancing their self‐renewal capacity, chemoresistance, and resistance to anoikis. Of particular significance, this investigation revealed a specific interaction between COPS3 and ITGB2, demonstrating that ITGB2 activation promotes COPS3 nuclear translocation. Subsequently, COPS3 maintains SOX2 protein stability by inhibiting its ubiquitin‐mediated degradation and enhances its transcriptional activity, thereby forming a positive feedback loop that reinforces the stemness phenotype. Furthermore, we discovered that SOX2 can form biomolecular condensates through LLPS and demonstrated the critical role of SOX2‐mediated LLPS in maintaining osteosarcoma stemness. These findings provide novel insights into the molecular regulation of osteosarcoma stemness and establish experimental evidence for developing targeted therapeutic strategies.

SOX2, a member of the HMG transcription factor family, plays a central role in maintaining pluripotency in embryonic stem cells and regulating early development. Recent studies have established its critical functions in CSCs across various cancer types, where it is implicated in tumor initiation, progression, and stemness maintenance [29, 30, 31, 32]. Although the expression and functional significance of SOX2 in osteosarcoma stem cells have been preliminarily confirmed, its precise regulatory mechanisms require systematic elucidation [33, 34, 35]. This study first identified SOX2 as a key downstream effector molecule of COPS3 in osteosarcoma, confirming that COPS3 maintains its protein stability by inhibiting the ubiquitin‐mediated degradation of SOX2. Although the regulatory role of the COP9 signalosome in the ubiquitin‐proteasome pathway has been documented [36, 37], the direct regulation of transcription factor stability, particularly SOX2, by specific subunits such as COPS3 remains unelucidated in osteosarcoma. Through molecular docking, Co‐IP, and protein degradation kinetics experiments, this study validated the interaction between COPS3 and SOX2, demonstrating that this interaction significantly delays the degradation of the SOX2 protein. These findings provide novel insights into the post‐translational regulatory mechanisms of key transcription factors in osteosarcoma stem cells. It should be noted that the E3 ligase mediating SOX2 ubiquitination remains unidentified. Further elucidating the regulatory relationship between COPS3 and potential E3 ligases represents a crucial direction for future research. Additionally, we observed that SOX2 binds to the COPS3 promoter region and enhances its transcriptional activity, suggesting the existence of a positive feedback regulatory loop between them, which may represent a crucial mechanism for maintaining high stemness in tumor cells.

On the other hand, this study established a connection between extracellular microenvironmental signals and intracellular stemness regulatory mechanisms. ITGB2, a key integrin family member, shows aberrant expression and activation in osteosarcoma [38, 39]. It indirectly regulates SOX2 transcriptional activity by promoting COPS3 nuclear translocation, thereby maintaining tumor cell stemness. This finding provides new molecular‐level insights into the interaction between CSCs and the ECM, suggesting that ITGB2 may play a crucial role in transducing extracellular microenvironmental signals into stemness‐maintaining signaling pathways. However, the precise mechanisms underlying the upregulated expression of ITGB2 in osteosarcoma cells remain elusive. Whether this results from paracrine signaling by immune cells within the tumor microenvironment or stems from intrinsic reprogramming of tumor cells requires further investigation. Elucidating this mechanism will potentially uncover novel pathways of tumor microenvironment interactions in stemness maintenance and provide potential therapeutic targets for cancer stemness‐directed strategies.

Furthermore, this study provided experimental evidence supporting the role of SOX2 in transcriptional regulation through LLPS in osteosarcoma. As an emerging mechanism of gene expression regulation, LLPS represents a relatively unexplored area in cancer stem cell research. Through in vitro droplet formation assays, FRAP, and functional inhibition experiments, we confirmed that SOX2 forms biomolecular condensates via LLPS and demonstrated the essential role of this process in activating cancer stemness‐related gene expression. However, although we hypothesize that COPS3 may indirectly influence SOX2 LLPS by maintaining its protein stability, whether COPS3 directly participates in regulating SOX2 phase separation requires further experimental validation. Moreover, the specific molecular determinants governing SOX2‐mediated LLPS and its regulatory mechanisms remain to be fully elucidated. Further investigation in this direction will contribute to a more comprehensive understanding of LLPS functionality in malignant tumor initiation and progression.

Building upon these elucidated molecular mechanisms, this study employed structure‐based drug design to identify a small‐molecule inhibitor Z‐5891 targeting COPS3. In both in vitro and in vivo experiments, Z‐5891 demonstrated significant antitumor efficacy, effectively suppressing osteosarcoma growth and reducing cancer stemness characteristics. Furthermore, Z‐5891 exhibited favorable biosafety profiles and remarkable therapeutic effects, suggesting its promising translational potential as a candidate therapeutic agent for osteosarcoma. These results support further development and preclinical investigation of this small‐molecule inhibitor.

In summary, this study systematically elucidated the role of COPS3 as a key regulator of osteosarcoma stemness and established a novel hierarchical regulatory cascade progressing from the ITGB2‐COPS3‐SOX2 signaling axis to SOX2 LLPS. Our findings not only provide new theoretical foundations for understanding the molecular mechanisms of osteosarcoma stemness but also identify promising therapeutic targets and candidate drugs for osteosarcoma treatment.

## 4 Experimental Section

### 4.1 Cell Lines and Cell Culture

Human OS cell lines 143B (TCH‐C401, HyCyte, RRID: CVCL_2270) were purchased from HyCyte (China). Human OS cell lines KHOS‐240S (CL‐0688, Procell, RRID: CVCL_2544) and Saos‐2 (CL‐0202, Procell, RRID: CVCL_0548) were purchased from Procell (China). All cell lines (143B, KHOS‐240S, and Saos‐2) were authenticated by short tandem repeat (STR) profiling and confirmed to be free of mycoplasma contamination. 143B and KHOS‐240S cells were cultured in DMEM medium (C11995500BT, Gibco) containing 10% serum (10099‐141, Thermo Fisher Scientific) and 1% antibiotics (15140‐122, Thermo Fisher Scientific). Saos‐2 cell line was cultured in McCoy's 5A (PM150710, Procell) medium containing 15% serum and 1% antibiotics. All cell lines were cultured in a humidified incubator with 5% CO2 at 37°C.

### 4.2 ITGB2 Activation Assay

For ITGB2 activation, cells were treated with the specific ITGB2/CD18 agonist Leukadherin‐1 (MedChemExpress, HY‐15701) at a final concentration of 5 µM in complete culture medium at 37°C.

### 4.3 Construction of Stable Cell Lines, siRNA and Transfection

Stable knockdown cell lines were generated using the pHBLV‐U6‐MCS‐PGK‐PURO lentiviral vector expressing specific shRNAs, with control cells expressing a non‐targeting scrambled shRNA from the same vector. Stable overexpression cell lines were generated using the pHBLV‐CMV‐MCS‐EF1‐Puro lentiviral vector, with the empty vector serving as the control. HA‐tagged ubiquitin (P0485, Shanghaihewubiotechnologyco.LTD) was transiently transfected into 143B cells using Lipofectamine 3000 (L3000015, Thermo Scientific). The oligonucleotide sequences of COPS3 shRNA, ITGB2 shRNA and SOX2 shRNA are listed in the Table S4.

### 4.4 Western Blotting

Cells were washed with phosphate‐buffered saline (PBS; C10010500BT,Thermo Fisher), and proteins were extracted using protein extracts (Cell Signaling Technology, 9803) containing protease inhibitors and phosphatase inhibitors (78440, Thermo Fisher Scientific). Total protein concentration was measured using a BCA kit (PC0020, Solarbio), followed by dilution of the protein using 5X Protein Sampling Buffer (LT101, EpiZyme) and boiling at 99°C for 5 min. Ensuring that the amount of protein uploaded was consistent for each lane, the proteins were separated by electrophoresis gel using a sodium dodecyl sulfate‐polyacrylamide gel and then transferred to polyvinylidene difluoride membranes. The proteins were transferred to the polyvinylidene fluoride membrane. The membranes were incubated overnight with primary antibody in TBST for 20 min. The next day, the excess primary antibody was rinsed with TBST and then the secondary antibody was incubated at 37°C for 1 h. Finally, the blot was detected by chemiluminescence. Information on the antibodies used in this study is provided in Table S5.

### 4.5 Quantitative Real‐Time PCR (qPCR) Analysis

Cells were cultured to 80% confluence, dissociated with pancreatin, and rinsed with PBS. Total RNA was isolated using TRIzol reagent, followed by DNase I digestion to remove genomic DNA. Reverse transcription was carried out with PrimeScript RT Master Mix. qPCR was performed on a fluorescence quantitative PCR system using the SYBR Green qPCR kit. GAPDH was used as an internal reference to calculate the relative expression level of the target gene, and the experiment was repeated three times. Primer sequences are provided in Table S6.

### 4.6 RNA Sequencing

The transcriptome and analysis were conducted by OE Biotech Co., Ltd. (Shanghai, China). Total RNA from the cells was extracted using TRIzol reagent, and RNA purity and quantification were characterized using a NanoDrop 2000 spectrophotometer (ThermoScientific, USA), and RNA integrity was assessed using an Agilent 2100 Bioanalyzer (Agilent Technologies, Santa Clara, CA, USA) to assess RNA integrity. Transcriptome libraries were constructed using the VAHTS Universal V5 RNA‐seq Library Prep kit. The libraries were sequenced using the llumina Novaseq 6000 sequencing platform. Data were preprocessed for subsequent analysis. Hierarchical clustering analysis of differentially expressed genes (DEGs) was performed using R (v 4.3.3) to demonstrate the expression patterns of genes in different groups and samples. Subsequently, GO, KEGG enrichment analysis was performed on the DEGs for screening significantly enriched functional entries.

### 4.7 Transwell Assay

A quantity of cells was resuspended in serum‐free medium, adjusted for concentration, and added to 200 µl into Transwell chambers (3422; Corning). 500 µL of medium containing 10% serum was added to the lower layer, and the chambers were placed on top of the lower culture wells. The cells were placed in a cell culture incubator for 6 h. After fixation with 4% tissue cell fixative, the cells were stained with 0.1% crystal violet for 15 min and observed under the microscope.

### 4.8 Cell Adhesion Assay

Vitronectin (AF‐VMB‐220, PeproTech) and fibronectin (354008, Biocoat) were diluted to a working concentration of 10 µg/mL in PBS. Add 100 µl of the protein solution to each well of a 96‐well plate and incubate overnight at 4°C. The next day unbound protein was washed with PBS and blocked with BSA for 30 min. Subsequently, 20,000 cells were seeded in wells that had been pre‐coated with protein and incubated in a cell culture incubator for 1 h. After fixation with 4% tissue cell fixative, the cells were stained with 0.1% crystal violet for 15 min and observed under the microscope.

### 4.9 Anoikis Resistance Assay

Resistance to anoikis was assessed by comparing cell viability under suspension versus adherent conditions. Briefly, cells were harvested to prepare a single‐cell suspension. For the suspension group, 2 × 10^3^ cells per well were seeded into ultra‐low attachment 96‐well plates in complete growth medium. For the parallel adherent control group, the same number of cells were seeded into standard tissue culture‐treated 96‐well plates. After incubation for 24 h at 37°C with 5% CO_2_, cell viability in both groups was quantified simultaneously using the Cell Counting Kit‐8 (CK04, DOJINDO). The relative survival rate was calculated as follows: (Luminescence of suspension group) / (Luminescence of adherent control group) × 100%.

### 4.10 Limiting Dilution Sphere Formation Assay

The self‐renewal capacity of cancer stem cells was assessed using a limiting dilution sphere formation assay. Briefly, a single‐cell suspension was obtained by filtering digested cells through a 40 µm cell strainer. Cells were then seeded in ultra‐low attachment 96‐well plates (3474, Corning) at clonal densities of 1, 5, 20, and 100 cells per well, with 24 replicate wells per density. The cells were cultured in serum‐free stem cell medium, which consisted of DMEM/F12 (11320033, Gibco) base medium supplemented with B27 (12587010, Gibco), human EGF (20 ng/mL, AF‐100‐15, PeproTech), and FGF basic (10 ng/mL, AF‐100‐18B, PeproTech). The plates were incubated at 37°C with 5% CO_2_ for 10–14 days. Half of the medium was gently replaced with fresh pre‐warmed medium every 3 days. Wells were examined under a microscope, and only wells containing perfectly round, phase‐bright spheres with a diameter ≥ 50 µm were scored as positive. The frequency of sphere‐initiating cells was calculated using Extreme Limiting Dilution Analysis (ELDA).

### 4.11 Co‐Immunoprecipitation

Proteins were extracted using protein extracts containing protease inhibitors and phosphatase inhibitors. Proteins in the Input group were added with 5X of sampling Buffer and boiled for 5 min at 99°C in a water bath and set aside. An appropriate amount of antibody to the target protein was added to the proteins of the experimental group and shaken at 4°C overnight. The next day pre‐treated Protein A/G agarose beads (20241, Thermo Scientific) were added to the proteins and shaken at 4°C for 1 h. The proteins were subsequently centrifuged at 4°C, 12,000 rpm for 15 min. The upper layer of liquid was discarded, and 5X of sampling buffer was added to the lower protein layer and boiled for 5 min at 99°C in a water bath. Detection was done using Western Blot.

### 4.12 Immunofluorescence Staining

An appropriate amount of well‐grown cells were inoculated onto 20 mm round slides and cultured until the next day until the cell confluence was about 70%. Cells were washed using PBS and fixed with 4% tissue cell fixative for 30 min. Cell permeabilization solution (P0097, Beyotime) was added and permeabilized for 20 min at room temperature. Primary antibody was added dropwise and incubated overnight at 4°C. Secondary antibody was added dropwise and incubated at 37°C for 1 h away from light. Subsequently, DAPI was added dropwise and incubated for 10 min away from light. The slides were blocked with anti‐fade mounting medium and the staining results were observed under fluorescence microscope.

### 4.13 Sirius Red and Masson's Trichrome Staining

For collagen fiber visualization, tissue sections were deparaffinized, rehydrated, and stained using standard protocols. For Sirius Red staining, sections were incubated in 0.1% Sirius Red F3B (G1078‐100ML, Servicebio) in saturated picric acid for 1 h, followed by rapid differentiation in 0.01 m HCl and dehydration. For Masson's Trichrome staining, sections were successively stained in Weigert's iron hematoxylin, Biebrich scarlet‐acid fuchsin, phosphotungstic acid, and aniline blue, according to the manufacturer's instructions (Masson Trichrome Stain Kit, G1006‐20ML, Servicebio). Stained sections were dehydrated, cleared, and mounted for bright‐field microscopy.

### 4.14 Immunohistochemical Staining

Tissue sections were deparaffinized by baking at 65°C, dewaxed by xylene and rehydrated by alcohol, and then passed through citrate buffer for high‐temperature repair of antigen. After serum closure, primary antibody was added dropwise and incubated overnight at 4°C, and the next day secondary antibody was added dropwise and incubated for 30 min at 37°C and washed to remove excess antibody. Next, endogenous peroxidase was blocked using 3% H2O2, followed by the addition of SABC and incubation at 37°C for 20 min. The sections were stained using DAB and stained again using hematoxylin. Finally, the stained sections were dehydrated, cleared and sealed, and the target protein expression was observed under the microscope.

### 4.15 ChIP‐PCR Assay

Cells were cultured to a certain abundance (number of cells not less than 1×107), and cell cross‐linking was performed by adding the appropriate concentration of formaldehyde to stabilize the interaction between protein and DNA. At the end of cross‐linking, glycine solution was added to terminate the cross‐linking reaction. Cells were then collected and washed with PBS. Cell membranes were lysed on ice using a cell lysis solution containing protease inhibitors to release chromatin from the nucleus. Chromatin was fragmented by sonication into fragments suitable for immunoprecipitation. The fragmented chromatin was diluted with dilution buffer and SOX2 antibody was added and incubated overnight at 4°C to bind the antibody to the target protein‐DNA complex. Protein A/G magnetic beads were added and incubation was continued at 4°C to bind the antibody‐target protein‐DNA complex to the magnetic beads. Multiple washes of the complexes were performed, followed by elution of the immunoprecipitated chromatin. NaCl, proteinase K were added and mixed and heated in a metal bath to promote uncrosslinking, followed by purification of the DNA fragments. The enriched DNA fragments were analyzed by quantitative PCR to detect the degree of enrichment of specific DNA sequences. The primers used for ChIP‐PCR are listed in Table S7.

### 4.16 Apoptosis Detection

Apoptosis in cells treated with small‐molecule inhibitors was assessed using Annexin V‐FITC/PI double staining (AP101, Multi Sciences) combined with flow cytometry. Apoptotic cells were quantified using a Beckman CytoFLEX flow cytometer, and apoptosis rates were determined with FlowJo software.

### 4.17 Protein Structure Acquisition and Protein‐Protein Docking

Obtained protein information via Uniprot (Universal Protein) and predicted protein structure using AlphaFold [40]. Protein‐protein docking was performed using the HDOCKlite v1.1 local version of the server and docking scores and confidence scores were calculated [41, 42, 43, 44]. The free energy of binding between proteins was calculated using MM/GBSA of HawkDock server. The binding interfaces of protein‐protein complexes were fully characterized and systematically analyzed using the PLIP interaction analysis platform. Interaction‐related details were then supplemented by pyMOL [45].

### 4.18 Fluorescence Recovery After Photobleaching (FRAP)

Cells were maintained in DMEM containing 10% FBS and seeded in glass‐bottom dishes (BS‐20‐GJM, Biosharp). When cells reached 70% confluence, the pcDNA3.0‐EGFP‐SOX2‐3×FLAG plasmid was transfected into cells using Lipofectamine 3000 Transfection Reagent. The final plasmid concentration was 1 mg/ml. Twenty‐four hours post‐transfection, cells were imaged using confocal microscopy. FRAP experiments were performed using a Zeiss LSM 980 microscope equipped with a 63× oil immersion objective. FRAP experiments were performed on regions within cells expressing fluorescently labeled proteins that were identical in area and shape. Droplets were bleached using a 488 nm laser (80% intensity, 1‐s dwell time), and the recovery rate of photobleaching was recorded over 2 min.

### 4.19 Extracellular Protein Imaging

To investigate the in vitro phase separation behavior of the SOX2 protein, a commercially available recombinant full‐length human SOX2 protein (Sino Biological, Cat: 13890‐H07E) was employed. First, the protein was co‐incubated and labeled with Alexa Fluor 488 NHS ester (Thermo Fisher Scientific, A20100). Subsequently, phase separation reactions were induced in an optimized buffer system within glass‐bottomed culture dishes. All samples were imaged using a Zeiss LSM 980 super‐resolution confocal microscope. To systematically evaluate phase separation characteristics, the following experiments were conducted: analyzing concentration dependence by adjusting protein concentration; adding 5% 1,6‐hexanediol to verify droplet dynamics; and capturing fusion and fission processes through time‐lapse imaging. Finally, ImageJ software was used for quantitative analysis, extracting parameters including droplet count, projected area, circularity, and average fluorescence intensity.

### 4.20 Virtual Screening

The target protein structure (PDB ID: 8H38) was preprocessed using the Protein Preparation Wizard module in Schrödinger software. This included preserving nucleic acid components, bond order optimization, hydrogenation, disulfide bond assignment, and protonation via the PROPKA method at pH 7.0. Subsequently, constrained energy optimization was performed using the OPLS4 force field, converging the heavy‐atom RMSD to 0.3 Å and optimizing side‐chain conformations to obtain a reliable protein structure. Small‐molecule ligands were sourced from the Hit Locator Library (approximately 460,000 compounds) within the Enamine database. Processed by the LigPrep module, they generated possible protonated states and tautomers at pH 7.0 ± 2.0 while preserving original chirality. Up to 32 conformations were generated per molecule to cover its conformational space. Preliminary screening was conducted using molecular docking methods, with compounds ranked based on docking scores. Further selection involved protein‐ligand interaction fingerprint analysis and clustering analysis to identify the highest‐scoring compounds in each category. The final selection of high‐potential candidate molecules was completed through manual evaluation [46, 47].

### 4.21 Inhibition Rate and IC50 Assay of Small‐Molecule Inhibitors

Seed 143B, KHOS and Saos‐2 cells in logarithmic growth phase at a density of 2200 cells per well in a 96‐well plate, dispensing 70 µL per well. Incubate overnight to allow complete attachment. Prepare a series of compound working solutions through serial dilutions, maintaining a consistent final DMSO concentration of 0.1%. Following cell attachment, 30 µL of the corresponding compound working solution was added to each well, and cultures were maintained for 72 h. After treatment, plates were equilibrated to room temperature. Each well received 100 µL of CTG reagent for cell lysis. After gentle shaking, samples incubated at room temperature for 10 min before chemiluminescence signals were detected using a microplate reader. Inhibition rates and IC50 values were calculated.

### 4.22 Surface Plasmon Resonance Detection

First, the COPS3 protein was immobilized as a ligand on a streptavidin (SA) chip, with the capture amount controlled at approximately 10,000 RU. To correct for solvent effects, buffers containing 4.5% and 5.8% DMSO were prepared, and corresponding calibration curves were established. During screening, 15 test compounds were diluted to concentrations of 50 µM, 5 µM, and 0 µM, with 50 µM acetazolamide (AAZ) serving as a negative control. Experiments were conducted in PBS buffer (pH 7.4) containing 0.05% Tween‐20 and 5% DMSO. Binding and dissociation times were set to 60 s each, with a wash time of 240 s and a flow rate of 30 µL/min. Data acquisition commenced after the system stabilized for 2.5 h at 25°C. All reagents used in the runs were filtered and degassed to ensure detection accuracy and reproducibility.

### 4.23 Molecular Docking and Molecular Dynamics Simulation

First, the CSN3 protein structure (PDB ID: 8H38) was retrieved from the PDB database. The binding pocket was predicted using the SiteFinder module in MOE software, with key residues including LEU138, LEU162, VAL164, ASP165, and MET166. The protein structure was optimized using MOE's Quickprep module, including hydrogenation, side‐chain repair, and protonation state adjustment, with the Amber10:EHT force field. Small molecules underwent energy minimization and multi‐conformation generation using the same module, with the optimal conformation selected for subsequent docking. During docking, based on the aforementioned binding site, the MOE‐DOCK module was employed: initial screening using London ΔG retained 300 conformations, followed by precise scoring with GBVI/WAS ΔG. The top 5 highest‐scoring conformations per molecule were ultimately selected for binding mode analysis. The optimal docking pose (Pose 1) was selected as the initial structure. The Desmond program was used to construct a cubic water box (TIP3P water model) with a boundary distance of 10 Å. Sufficient Na^+^/Cl^−^ ions were added to neutralize charges, maintaining a salt concentration of 0.15 mol/L. The system underwent energy minimization with the protein backbone constrained (1 kcal/mol/Å^2^). followed by heating from 0 to 300 K under NVT ensemble conditions (100 ps), then density equilibrium under NPT ensemble (100 ps). Finally, a 100 ns molecular dynamics simulation was conducted with temperature and pressure controlled by Nose‐Hoover and MTK methods, respectively, at 300 K and 1 atm pressure. The cutoff radius was set to 9 Å, and the time step was 2 fs.

### 4.24 Single‐Cell RNA Sequencing Analysis

This study analyzed single‐cell RNA sequencing datasets from the public databases GSE152048, GSE162454, and GSE250015. All data preprocessing and fundamental analyses were performed using the Seurat software package (v4.0). To integrate batch effects across different data sources, batch correction was conducted using the Harmony software package. Differentially expressed genes were identified via the FindMarkers and FindAllMarkers functions, with screening thresholds set as: adjusted p‐value < 0.05, log_2_ fold change > 0.25, and expression proportion > 10%. Gene expression patterns were visualized using VlnPlot and FeaturePlot. Heatmaps were generated with the pheatmap package, and overall figures were created using ggplot2. ClusterProfiler was further employed for GO and KEGG functional enrichment analysis, as well as gene set enrichment analysis (GSEA) of differentially expressed genes. Copy number variation (CNV) inference was performed using copykat software with the following parameters: id.type = “S”, ngene.chr = 5, win.size = 25, KS.cut = 0.1, reference genome hg20, and 40 parallel threads. Based on the prediction results, cells were classified into “aneuploid” and “euploid” categories, and the corresponding copy number variation matrix was extracted. Pathway activity in cell subpopulations was assessed using the GSVA method, employing gene sets from the Hallmark collection in MSigDB. Tumor cells were further divided into COPS3‐positive and COPS3‐negative subpopulations based on COPS3 gene expression levels. Immune‐related gene set analysis employed the irGSEA tool, integrating four algorithms—AUCell, UCell, singscore, and ssgsea—to evaluate pathway activity based on MSigDB Hallmark gene sets. The Wilcoxon rank‐sum test identified significantly different pathways (adjusted p‐value < 0.05), and the RobustRankAggreg method aggregated multi‐algorithm results to visualize pathways significant in at least three methods. Cell pluripotency was assessed using both CytoTRACE2 and SCENT algorithms. CytoTRACE2 inferred cellular differentiation states from gene expression matrices, with results visualized in UMAP plots alongside cell phenotypes. The SCENT algorithm calculated pluripotency indices by integrating the protein interaction network (net17Jan16.m) and inferred developmental trajectories through diffusion mapping. All data analysis and statistics were performed in R 4.3.3, Python 3.10, and the Ubuntu operating system to ensure consistency and reproducibility of the analytical process.

### 4.25 Animal Models and Therapeutics

Animal experiments were conducted according to the protocol approved by the Animal Ethics Committee of Peking University People's Hospital (Reference No.: 2023PHE096). A 100 µL suspension containing 2×10^6^ 143B cells was implanted via subcutaneous injection into the right scapular region of five‐week‐old Balb/c nude mice. When tumor volume reached approximately 100 mm^3^, tumor‐bearing mice were randomly divided into four groups (n = 8) and treated for 2 weeks with: equal volume solvent (tail vein injection every other day), Z‐5891 (tail vein injection every other day, 20 mg/kg), DOX (tail vein injection twice weekly, 3 mg/kg), or Z‐5891 + DOX. During this period, the longest diameter (L) and shortest diameter (W) of the tumors were measured every 3 days using a Vernier caliper. Tumor volume was calculated using the formula (L×W^2^) / 2 to plot tumor growth curves. At the end of the treatment cycle, all mice were euthanized, and tumor tissues were surgically excised and weighed for subsequent analysis.

### 4.26 Rationale for Z‐5891 Dosage Selection

The in vivo dosage of Z‐5891 (20 mg/kg) was selected based on a comprehensive preclinical evaluation. First, the in vitro anti‐proliferative activity of Z‐5891 against 143B cells was determined, providing a baseline for in vivo potency. Subsequently, a dose‐finding study was performed in tumor‐bearing mice. Animals were administered Z‐5891 at doses of 5, 10, 20, and 40 mg/kg (tail vein injection, every other day) for two weeks. This pilot study identified 20 mg/kg as the optimal dose, which effectively suppressed tumor growth without causing significant toxicity, as evidenced by stable body weight and normal serum biochemical profiles.

### 4.27 Human Tissues

The OS specimens used in this study were obtained from the Musculoskeletal Tumor Center of Peking University People's Hospital (Beijing, China). The study was approved by the Ethics Committee of Peking University People's Hospital and informed consent was obtained from all patients (2019PHB198‐01).

### 4.28 Statistical Analysis

Differences between groups were analyzed using unpaired t‐tests, one‐way ANOVA, and two‐way ANOVA, with p < 0.05 set as the threshold for statistical significance. All statistical analyses were performed using GraphPad Prism. * indicates p < 0.05; ** indicates p < 0.01; *** indicates p < 0.001; **** indicates p < 0.0001.

## Funding

This work was supported by the National Natural Science Foundation of China (82573696, 82141120, 82372739 and 81972513), Natural Science Foundation of Beijing, China (7232186), and National High Level Hospital Clinical Research Funding (Interdisciplinary Research Project of Peking University First Hospital, 2024IR43).

## Conflicts of Interest

The authors declare that they have no competing interests.

## Author Contributions

Lei Guo: Conceptualization, Project administration, Supervision, Validation, Visualization, Writing – original draft, Writing – review & editing. Zhiqing Zhao: Writing – review & editing. Wei Wang: Writing – review & editing. Jianfang Niu: Methodology. Bing Wang: Validation. Yunfei Lin: Visualization. Wei Guo: Supervision. Taiqiang Yan: Conceptualization, Validation, Visualization, Writing – original draft, Writing – review & editing.

## Supporting information




**Supporting File**: advs74798‐sup‐0001‐SuppMat.pdf.

## Data Availability

The data that support the findings of this study are available from the corresponding author upon reasonable request.
