# Supplementary material for: The ITGB2‐COPS3‐SOX2 Axis and SOX2 Liquid‐Liquid Phase Separation: Dual Mechanisms Governing Osteosarcoma Stemness
Source: Adv Sci (Weinh). 2026 Mar 13;13(29):e20913. doi: 10.1002/advs.202520913 (PMC13205827; doi:10.1002/advs.202520913)
Supplement: Supplementary file 1 — Supporting File: advs74798‐sup‐0001‐SuppMat.pdf. [file ADVS-13-e20913-s001.pdf]

Supporting Information

The ITGB2-COPS3-SOX2 Axis and SOX2 Liquid-Liquid Phase Separation: Dual Mechanisms Governing Osteosarcoma Stemness

*Lei Guo, Zhiqing Zhao, Wei Wang, Jianfang Niu, Bing Wang, Yunfei Lin, Wei Guo and Taiqiang Yan**

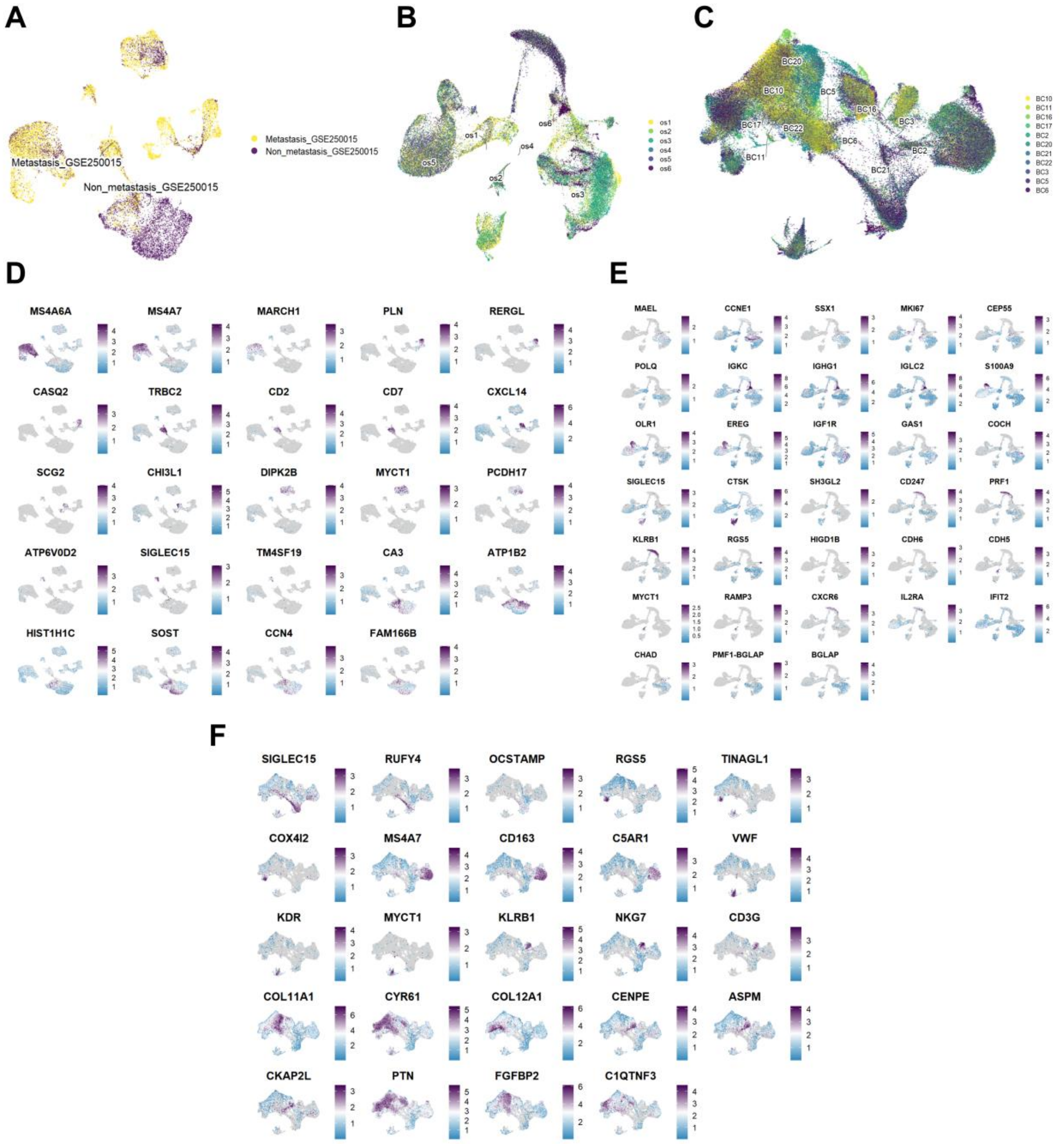

Figure S1. Dimension reduction UMAP plot and top marker gene feature plot of single-cell RNA sequencing datasets. (A) GSE250015 integrated dimension reduction UMAP plot. (B) GSE162454 integrated dimension reduction UMAP plot. (C)

GSE152048 integrated dimension reduction UMAP plot. (D) GSE250015 cell top marker gene feature plot. (E) GSE162454 cell top marker gene feature plot. (F) GSE152048 cell top marker gene feature plot.

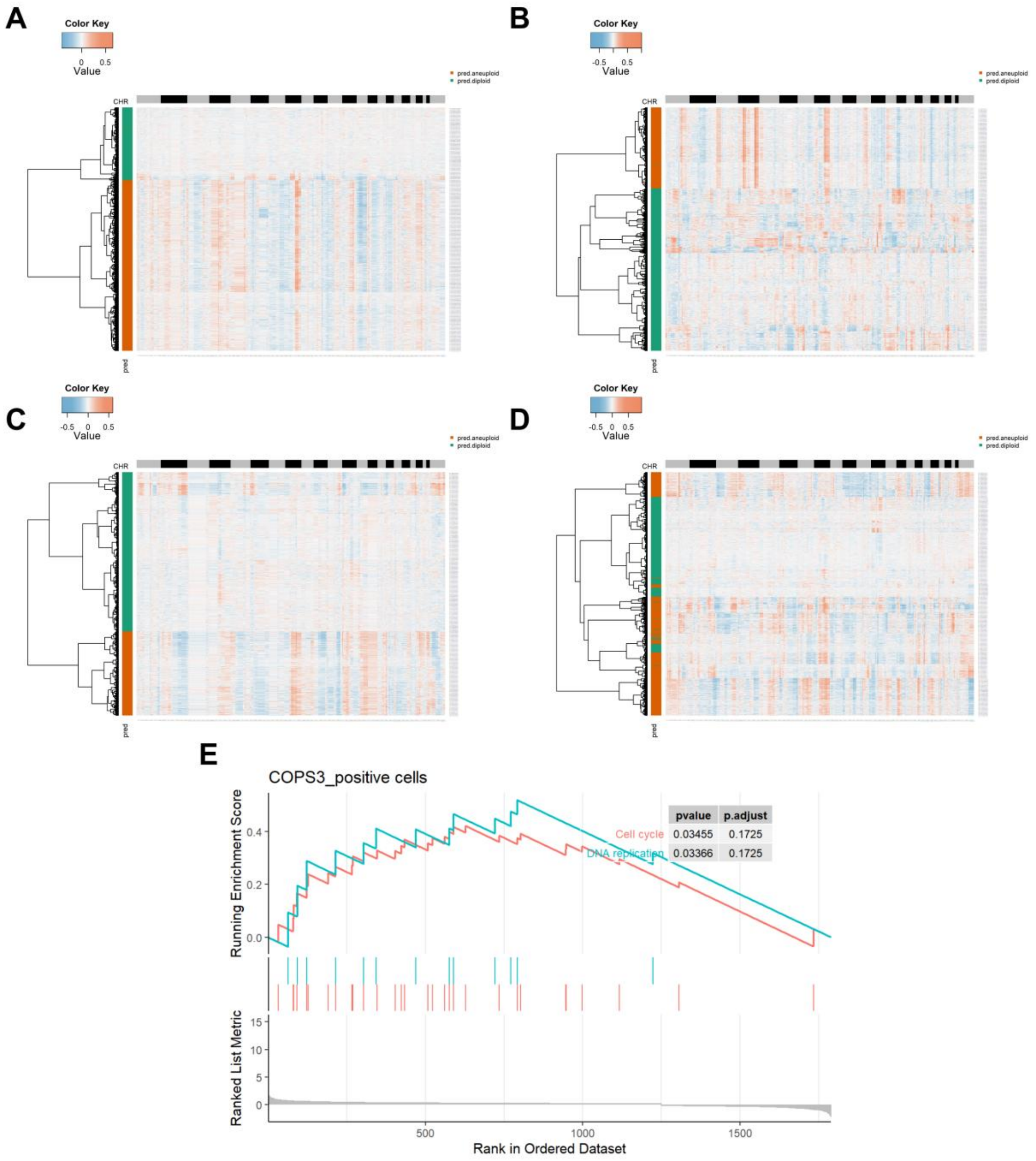

Figure S2. Single-cell RNA sequencing datasets tumor cell CNV analysis. (A) GSE250015 tumor cell CNV analysis. (B) GSE162454 tumor cell CNV analysis. (C-D) GSE152048 tumor cell CNV analysis. (E) COPS3+ cancer cell GSEA

enrichment plot.

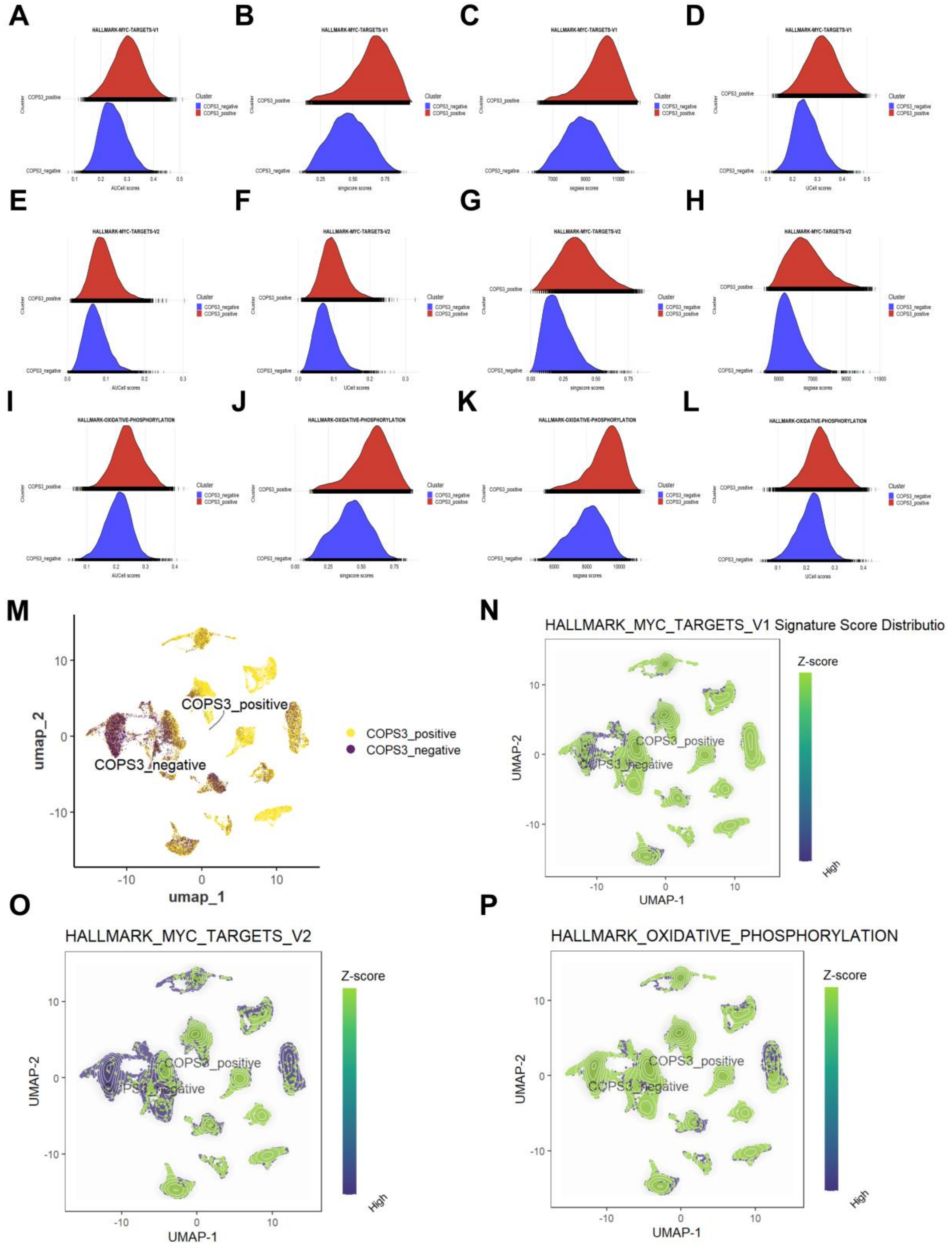

Figure S3. Single-cell pathway analysis ridge plot. (A-D) MYC-TARGET-V1 single-cell pathway analysis ridge plot. (E-H) MYC-TARGET-V2 single-cell pathway analysis ridge plot. (I-L) Oxidative phosphorylation single-cell pathway analysis ridge

plot. (M) Single-cell dimension reduction plot of cancer cells. (N) MYC-TARGET-V1 dimension reduction score plot. (O) MYC-TARGET-V2 dimension reduction score plot. (P) Oxidative phosphorylation dimension reduction score plot.

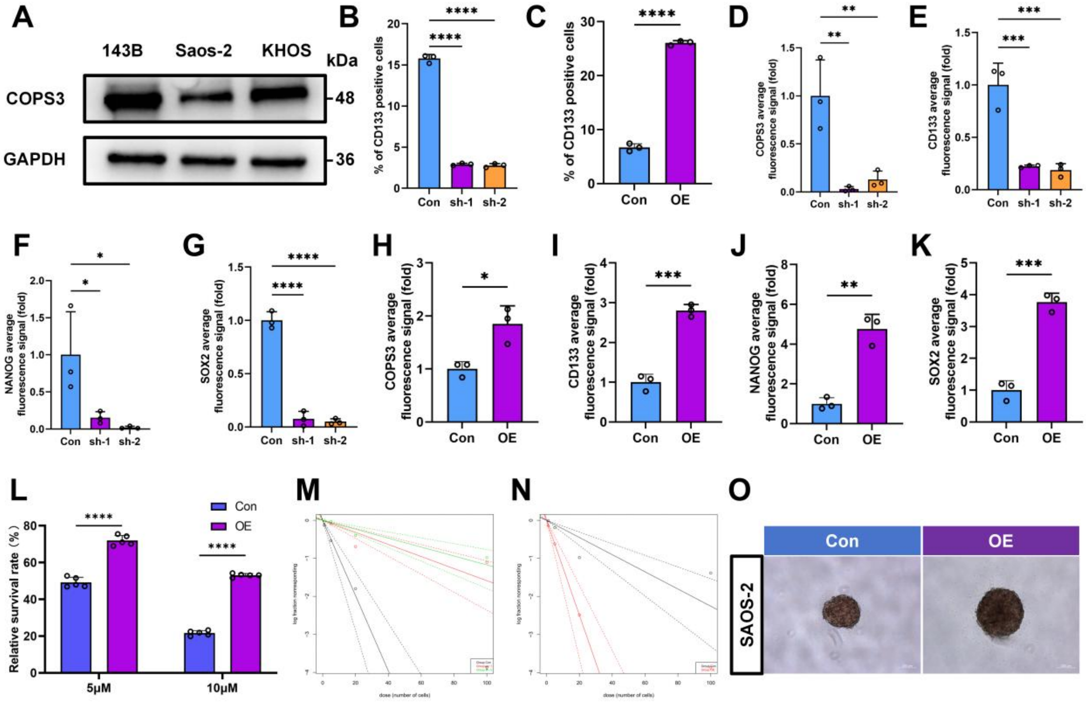

Figure S4. High expression of COPS3 maintains stemness in osteosarcoma cells. (A) Detection of COPS3 expression in different cell lines by Western blotting. (B-C) Effects of COPS3 knockdown (B) or overexpression (C) on CD133 expression in cells were detected by flow cytometry, n = 3. (D-G) Semi-quantitative results of the fluorescence intensity in Figure 2E, n = 3. (H-K) Semi-quantitative results of the fluorescence intensity in Figure 2F, n = 3. (L) Effect of overexpression of COPS3 on the ability of Saos-2 cells to resist doxorubicin, n = 5. (M) ELDA showing the estimated tumor-initiating cell frequencies for Control, COPS3-sh1, and COPS3-sh2 groups. (N-O) Effects of overexpression of COPS3 on the ability of cells to sphere

formation.

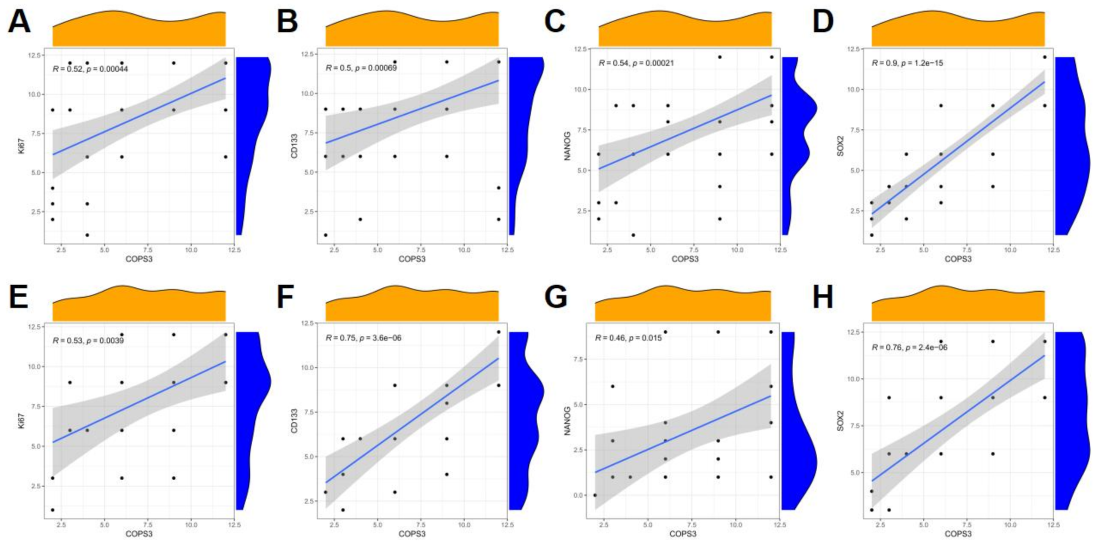

Figure S5. Correlation analysis of COPS3 and CSC-related markers expression in osteosarcoma tissues. (A-D) Correlation analysis of COPS3 and CSC-related markers expression in primary tissues of osteosarcoma. (E-H) Correlation analysis of COPS3 and CSC-related markers expression in osteosarcoma lung metastatic tissues.

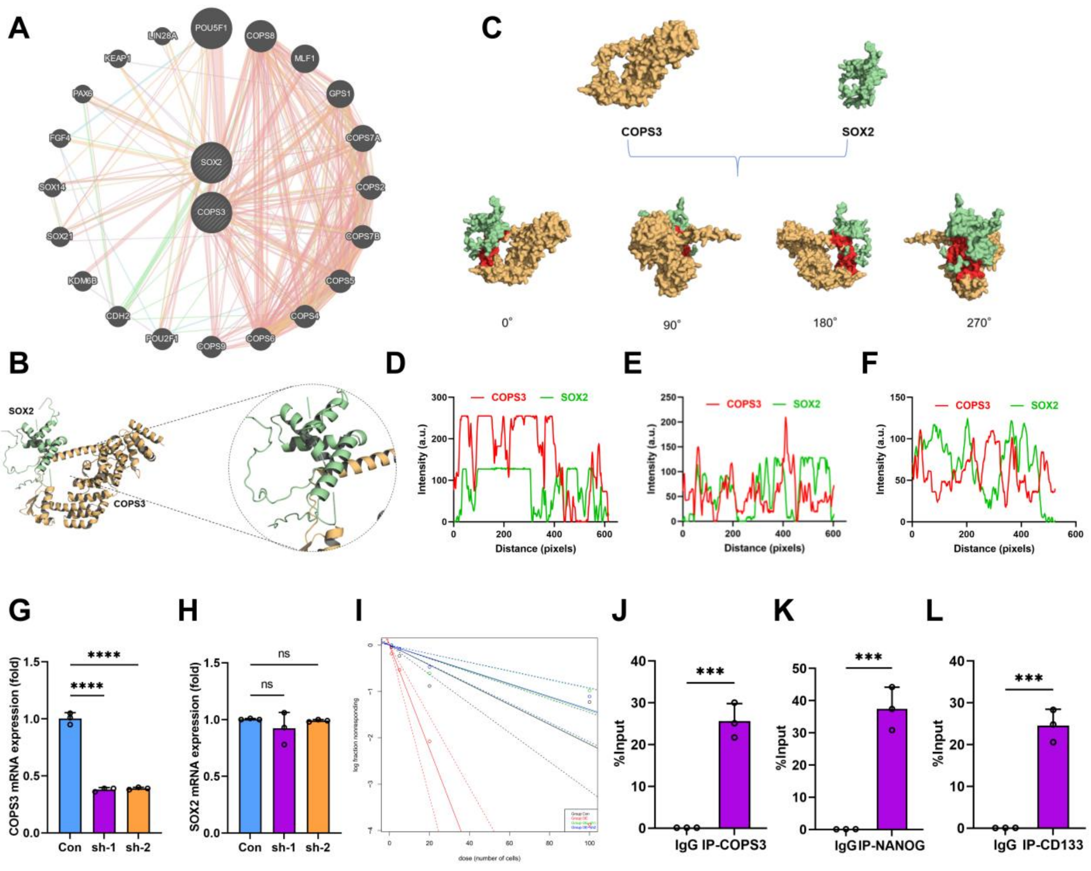

Figure S6. COPS3 regulates stemness in osteosarcoma cells by affecting SOX2. (A) Interaction analysis of COPS3 and SOX2. (B) HDOCK server docking result. COPS3: yellow; SOX2: green. (C) Surface form visualization of docking results. COPS3: yellow ; SOX2: green; Protein-to-protein contact area: red. (D-F) Colocalization analysis of COPS3 and SOX2 in 143B (D), KHOS (E), and Saos-2 (F) cells. COPS3: red; SOX2: green. (G) The effect of COPS3 knockdown on COPS3 mRNA expression levels in 143B cells was detected by qPCR. (H) The effect of COPS3 knockdown on SOX2 mRNA expression levels in 143B cells was detected by qPCR. (I) COPS3 affected the ability of Saos-2 cells to sphere formation by affecting SOX2. (J) ChIP assay of SOX2 interaction with COPS3 promoter in 143B cells. (K) ChIP assay of SOX2 interaction with NANOG promoter in 143B cells. (L) ChIP assay of SOX2

interaction with CD133 promoter in 143B cells.

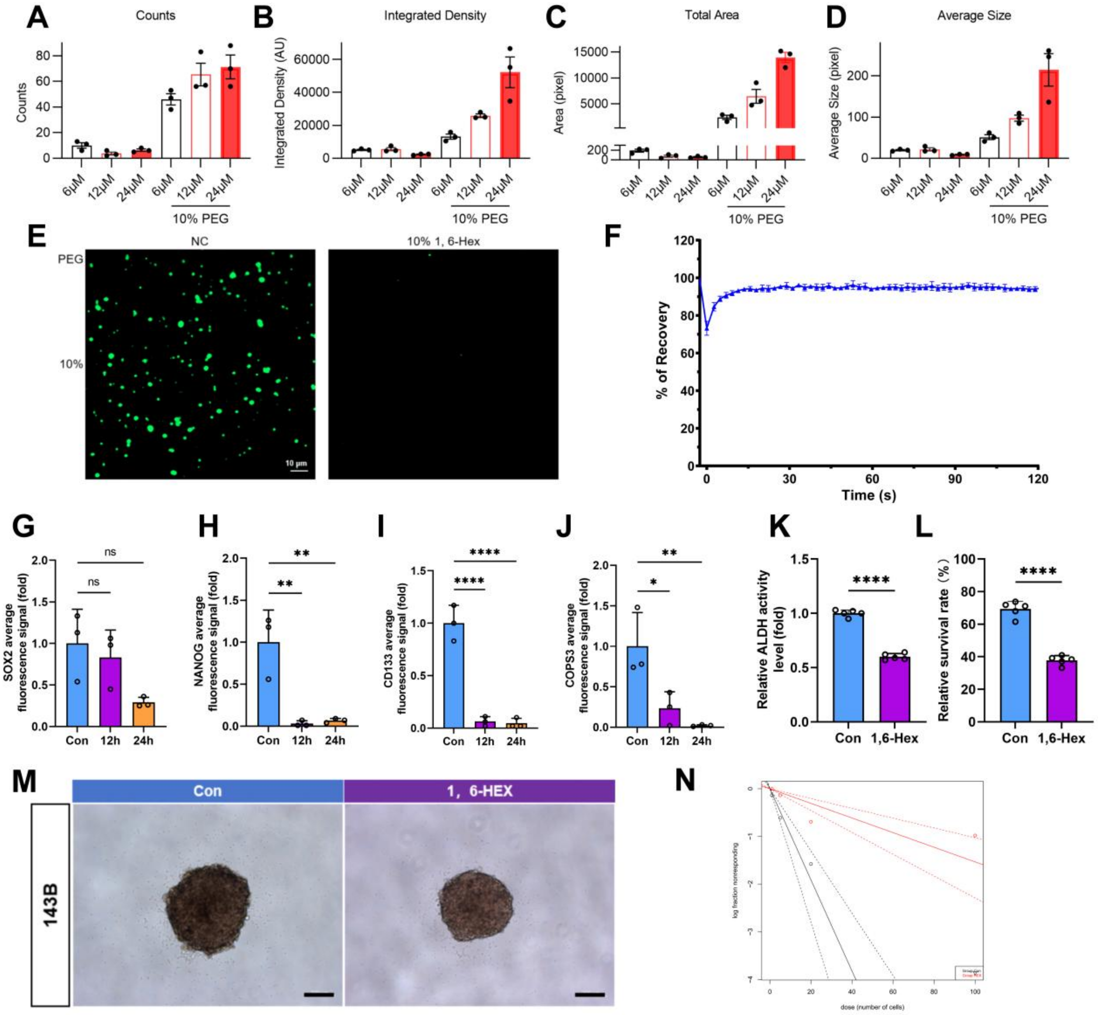

Figure S7. SOX2 regulates osteosarcoma cell stemness through liquid-liquid phase separation (LLPS) properties. (A-D) Statistical diagram of in vitro imaging of the SOX2 protein. (E) In vitro 1,6-hexanediol destruction of SOX2 protein imaging (scale bar, 10 μm). (F) Intracellular FRAP. (G-J) Semi-quantitative results of the fluorescence intensity in Figure 3Q, n = 3. (K) Effect of 1,6-Hexanediol treatment on ALDH activity in 143B cells, n = 5. (L) Effect of 1,6-Hexanediol treatment on the ability of 143B cells to resist anoikis, n = 5. (M-N) Effects of 1,6-Hexanediol treatment on the ability of cells to sphere formation (scale bar, 200 μm). Results are

reported as means ± SD. *P < 0.05, **P < 0.01, ***P < 0.001, ****P < 0.0001.

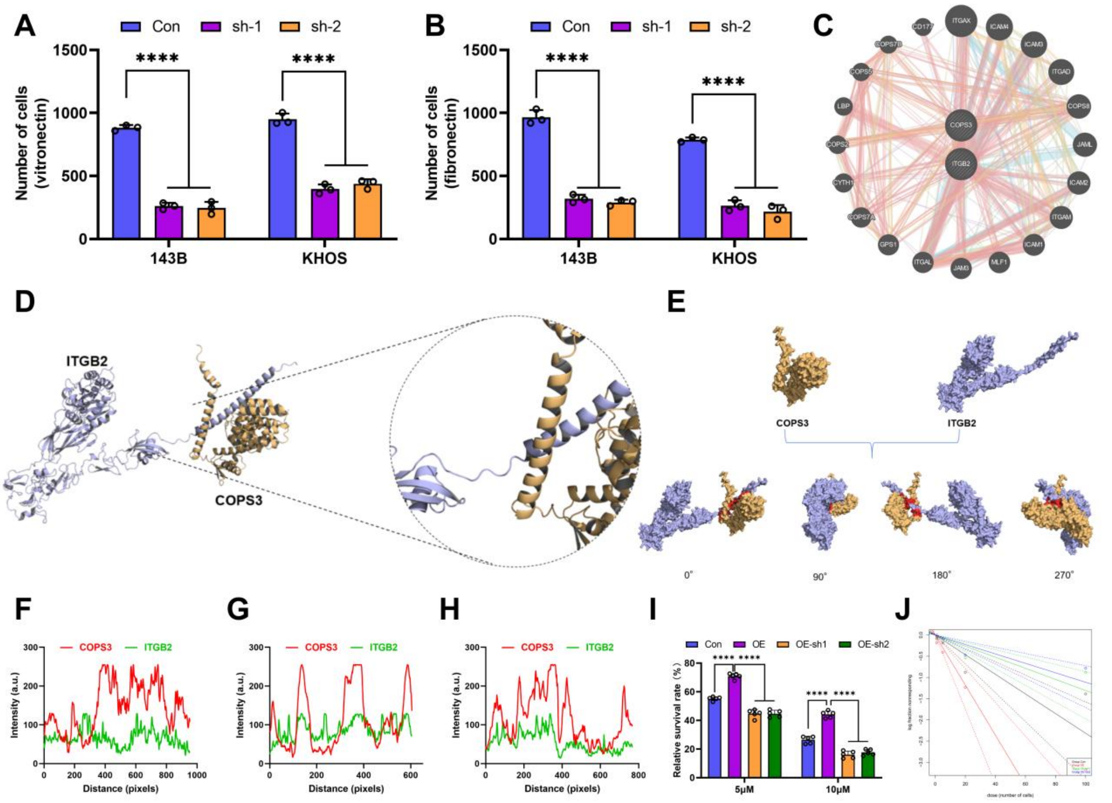

Figure S8. ITGB2 regulates stemness in osteosarcoma cells by affecting COPS3. (A-B) Quantitative analysis of the effect of knockdown of COPS3 on the ability of 143B and KHOS cells to adhere to vitronectin (A) and fibronectin (B), n = 3. (C) Interaction analysis of COPS3 and ITGB2. (D) HDOCK server docking result. COPS3: yellow; ITGB2: purple. (E) Surface form visualization of docking results. COPS3: yellow ; ITGB2: purple; Protein-to-protein contact area: red. (F-H) Colocalization analysis of COPS3 and ITGB2 in 143B (F), KHOS (G), and Saos-2 (H) cells. COPS3: red ; ITGB2: green. (I) ITGB2 affected the ability of Saos-2 cells to resist doxorubicin by affecting COPS3, n = 5. (J) ITGB2 affected the ability of Saos-2 cells to sphere formation by affecting COPS3. Results are reported as means ± SD.

****P < 0.0001.

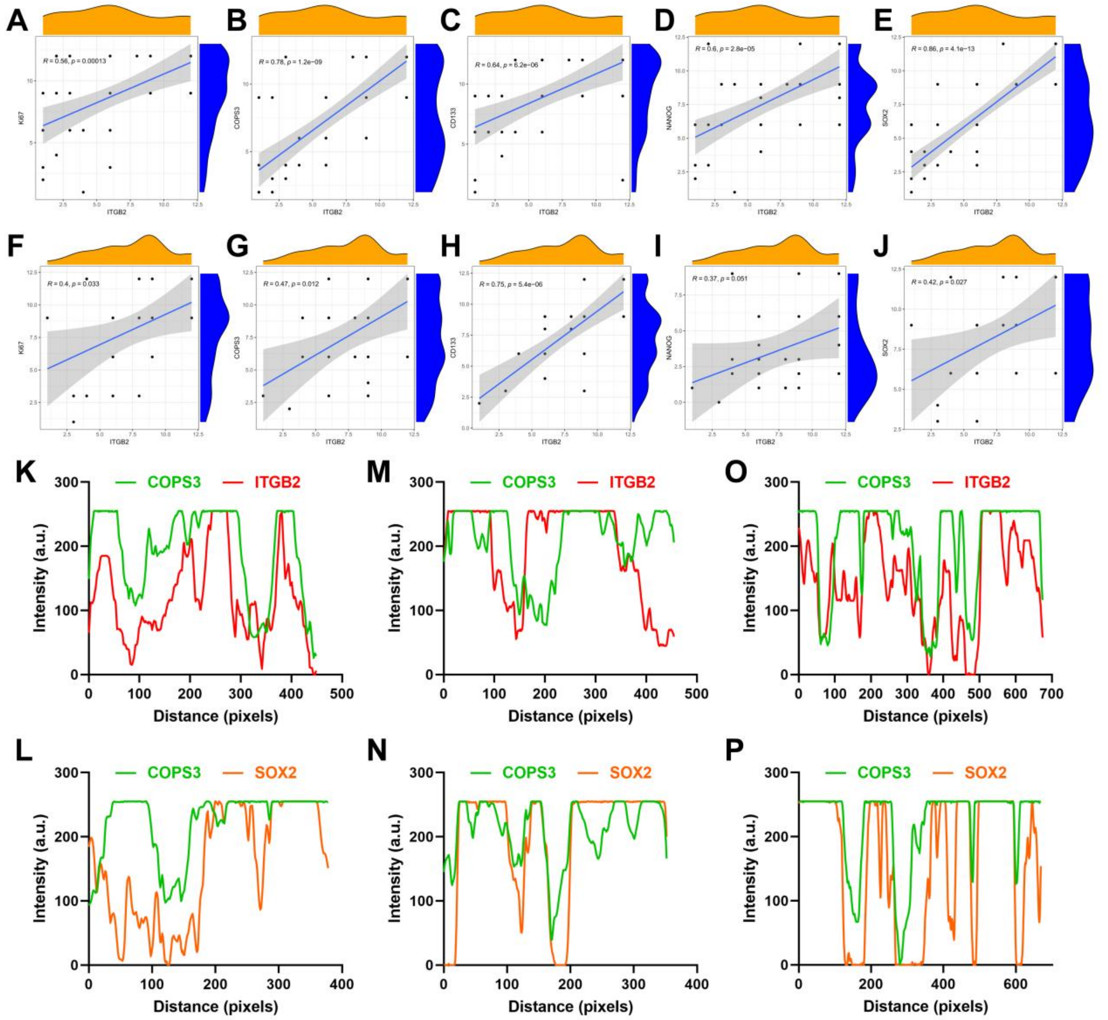

Figure S9. Correlation analysis of ITGB2 and CSC-related markers expression in osteosarcoma tissues. (A-E) Correlation analysis of ITGB2 and CSC-related markers expression in primary tissues of osteosarcoma. (F-J) Correlation analysis of ITGB2 and CSC-related markers expression in osteosarcoma lung metastatic tissues. (K-L) Significant colocalization was observed between ITGB2 and COPS3 (K), as well as between COPS3 and SOX2 (L), in clinical osteosarcoma specimens (Patient 1). (M-N) Significant colocalization was observed between ITGB2 and COPS3 (M), as well as between COPS3 and SOX2 (N), in clinical osteosarcoma specimens (Patient

2). (O-P) Significant colocalization was observed between ITGB2 and COPS3 (O), as well as between COPS3 and SOX2 (P), in clinical osteosarcoma specimens (Patient 3).

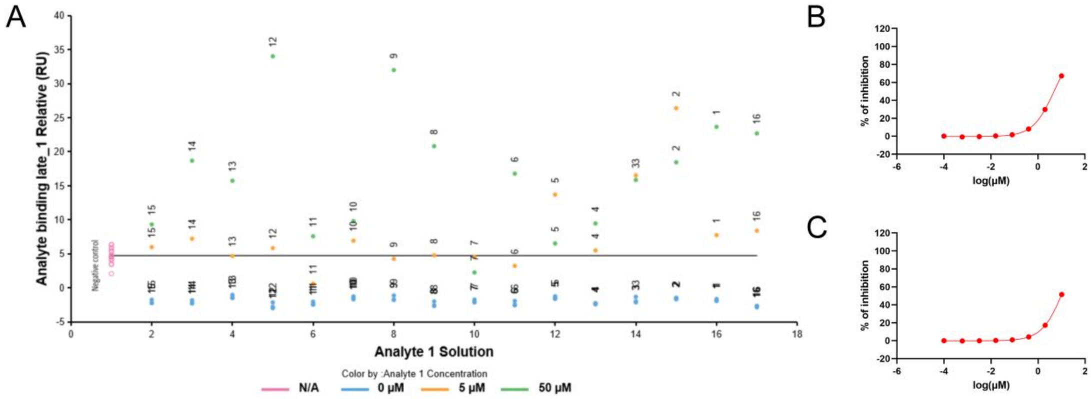

Figure S10. Detection of the interaction between 15 small molecules and COPS3 proteins via SPR. (A) Detection of the interaction between 15 small molecules and COPS3 proteins via SPR. (B) Z-5891 exhibits concentration-dependent proliferation-inhibitory activity against KHOS cells. (C) Z-5891 exhibits concentration-dependent proliferation-inhibitory activity against Saos-2 cells.

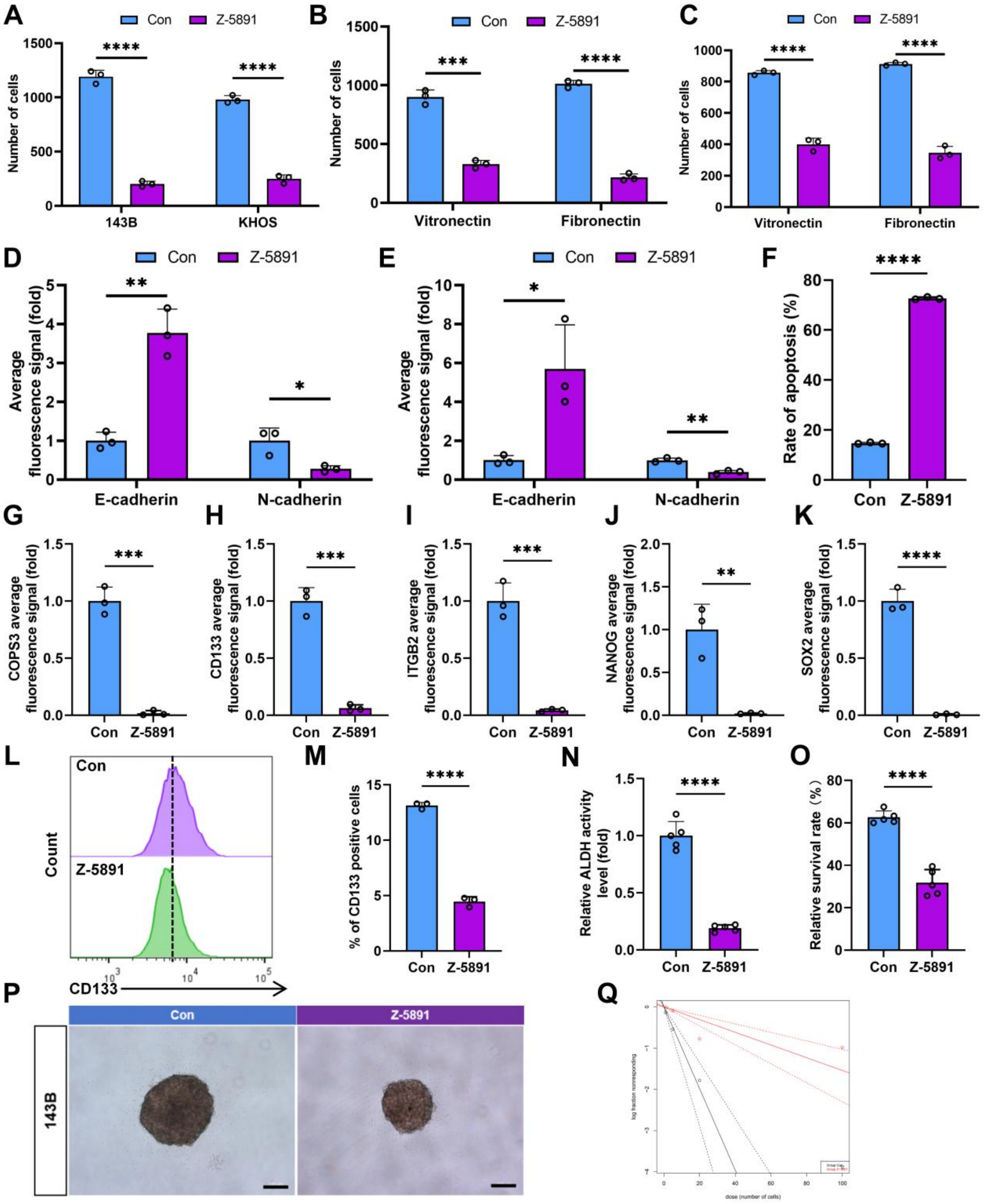

Figure S11. Z-5891 suppresses multiple malignant phenotypes in osteosarcoma cells. (A) Quantitative analysis of the effect of Z-5891 treatment on the invasive ability in 143B and KHOS cells, n = 3. (B-C) Quantitative analysis of the effect of Z-5891 treatment on the ability of 143B (B) and KHOS (C) cells to adhere to vitronectin and fibronectin, n = 3. (D) Semi-quantitative analysis of E-cadherin and N-cadherin immunofluorescence staining results in 143B cells, n = 3. (E) Semi-quantitative

analysis of E-cadherin and N-cadherin immunofluorescence staining results in KHOS cells, n = 3. (F) Quantitative analysis of the effect of Z-5891 treatment on apoptosis in 143B cells, n = 3. (G-K) Semi-quantitative results of the fluorescence intensity in Figure 6H, n = 3. (L-M) Effect of Z-5891 treatment on CD133 expression in 143B cells was detected by flow cytometry, n = 3. (N) Effect of Z-5891 treatment (3.28 μM) on ALDH activity in 143B cells, n = 5. (O) Effect of Z-5891 treatment (3.28 μM) on the ability of 143B cells to resist anoikis, n = 5. (P-Q) Effects of Z-5891 treatment (3.28 μM) on the ability of cells to sphere formation (scale bar, 200 μm). Results are reported as means ± SD. *P < 0.05, **P < 0.01, ***P < 0.001, ****P < 0.0001.

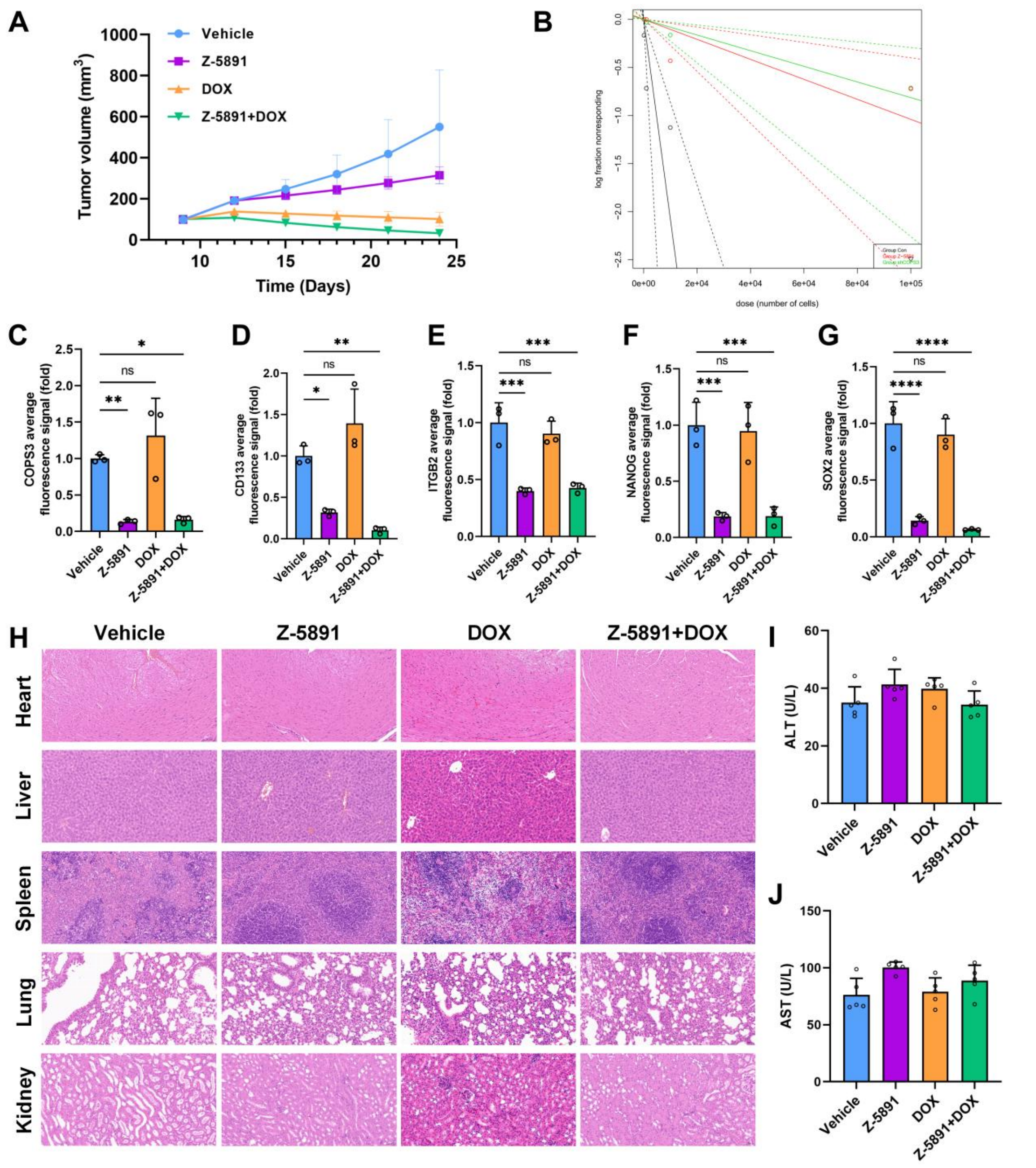

Figure S12. Biosafety testing of Z-5891. (A) Tumor growth curves for each group. (B) Extreme limiting dilution analysis showing the estimated tumor-initiating cell frequencies for Control, shCOPS3, and Z-5891 groups. (C-G) Semi-quantitative results of the fluorescence intensity in Figure 7E, n = 3. (H) Comprehensive H&E staining images depicting complete views of the main organs. (I-J) The level of ALT (I) and AST (J) in mouse after being treated with different drugs, n = 5. ALT, alanine aminotransferase; AST, aspartate aminotransferase. Results are reported as means ±

SD. *P < 0.05, **P < 0.01, ***P < 0.001, ****P < 0.0001.

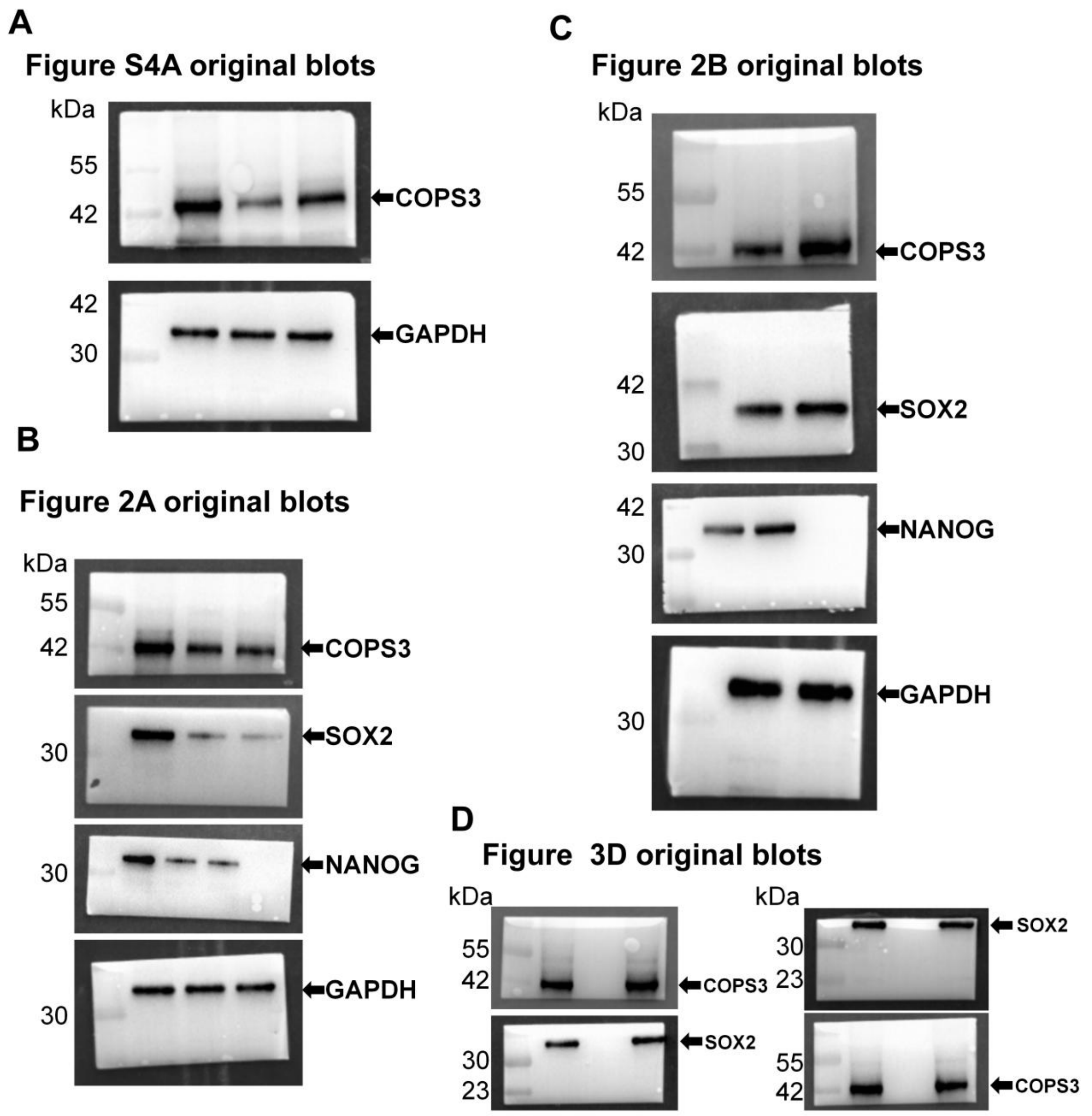

Figure S13. Original, unaltered images of blots found in this study. (A) Figure S4A original blots. (B) Figure 2A original blots. (C) Figure 2B original blots. (D) Figure 3D original blots.

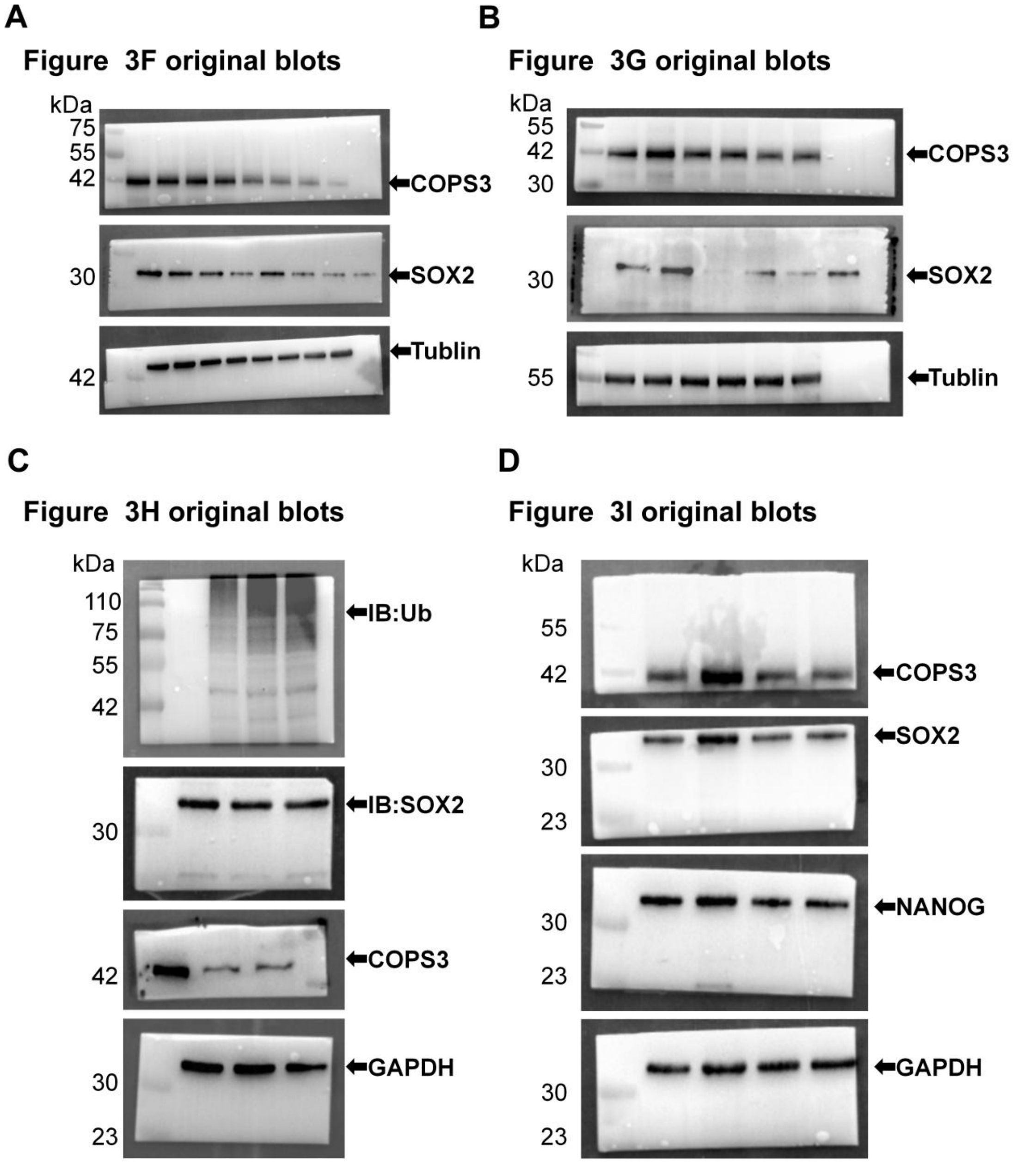

Figure S14. Original, unaltered images of blots found in this study. (A) Figure 3F original blots. (B) Figure 3G original blots. (C) Figure 3H original blots. (D) Figure 3I original blots.

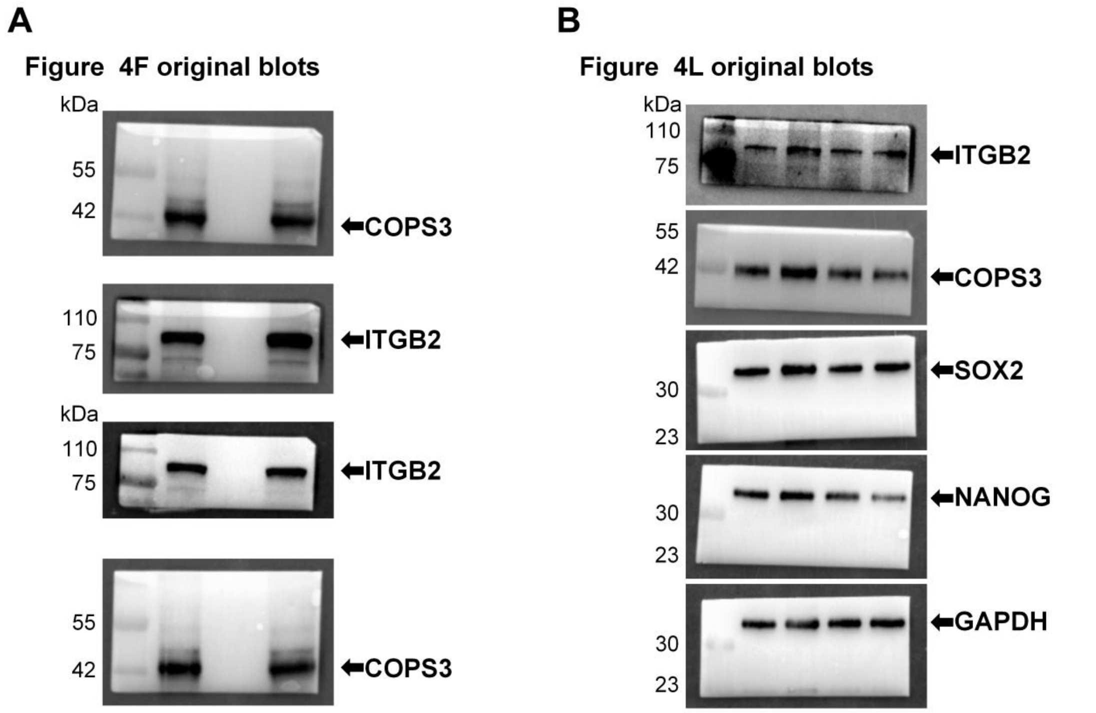

Figure S15. Original, unaltered images of blots found in this study. (A) Figure 4F original blots. (B) Figure 4L original blots.

Table S1. Differentially expressed transcription factors related to cancer stem cells.

ID	log2FoldChange	pvalue	padj	Description
**A2M**	-1.130304638	0.003410895	0.21341176	alpha-2-macroglobulin
**CITED1**	-0.588922067	0.018844346	0.429846866	Cbp/p300 interacting transactivator with Glu/Asp rich carboxy-terminal domain

				1
**DLL1**	-0.720239565	0.000185228	0.049549756	delta like canonical Notch ligand 1
**FGF19**	-1.261125642	0.006486147	0.282996733	fibroblast growth factor 19
**FGF2**	0.598850142	0.007548651	0.301079339	fibroblast growth factor 2
**GPM6A**	-0.726223334	0.037395648	0.551011334	glycoprotein M6A
**HES2**	-1.001616894	0.003004394	0.20177969	hes family bHLH transcription factor 2
**LBH**	-0.766946509	0.008549553	0.322320926	LBH regulator of WNT signaling pathway
**MEOX1**	-3.032883531	0.048089541	0.593038072	mesenchyme homeobox 1
**NOG**	-0.937564608	0.000219434	0.054063479	noggin
**NOTCH3**	-0.642688225	0.026267639	0.487275234	notch receptor 3
**NRTN**	-0.96665285	0.014684285	0.395600898	neurturin
**OSR1**	4.528760287	0.027573151	0.497784366	odd-skipped related transcription factor 1
**SEMA3C**	0.656313797	0.017573034	0.422486875	semaphorin 3C
**SEMA4B**	-0.822285891	0.00123378	0.137036184	semaphorin 4B
**SEMA5A**	-1.069519195	0.00542148	0.268440848	semaphorin 5A
**SP7**	-4.591899774	0.045200263	0.580452058	Sp7 transcription factor
**WNT6**	-1.820579737	0.002515231	0.19032582	Wnt family member 6

Table S2. Conformation affinity score.

**Pose**	**Docking Score**
1	-6.4678988
2	-6.3639998
3	-6.3442779
4	-6.3218565
5	-6.3045311

Table S3. COPS3 protein and Z-5891 interaction information.

Hydrophobic Interactions	Residue	Distance
1	ALA 178	3.97
2	PHE 181	3.69
3	TYR 203	3.95
4	PHE 204	3.38
**Hydrogen Bonds**		
1	MET 166	2.06
2	GLN 207	2.81
**π-Stacking**		
1	TYR 185	5

Table S4. The sequences of short hairpin RNA (shRNA).

shRNA	Forward	Reverse
COPS3 RNAi-1	GATCCGCACATTCGATATGCAACA TTCAAGAGATGTTGCATATCGAAT GTGCTTTTTTG	AATTCAAAAAAGCACATTCGATA TGCAACATCTCTTGAATGTTGCAT ATCGAATGTGCG
COPS3 RNAi-2	GATCCGGTTGATTGTTGTTTGCTAT TCAAGAGATAGCAAACAACAATC AACCTTTTTTG	AATTCAAAAAAGGTTGATTGTTGT TTGCTATCTCTTGAATAGCAAACA ACAATCAACCG
ITGB2 RNAi-1	GATCCGAAACCCAGGAAGACCAC AATCTCGAGATTGTGGTCTTCCTG GGTTTCTTTTTTG	AATTCAAAAAAGAAACCCAGGAA GACCACAATCTCGAGATTGTGGTC TTCCTGGGTTTCG
ITGB2 RNAi-2	GATCCGCTCAAGTCCCAGTGGAAC AATGATCTCGAGATCATTGTTCCA CTGGGACTTGAGCTTTTTTG	AATTCAAAAAAGCTCAAGTCCCA GTGGAACAATGATCTCGAGATCA TTGTTCCACTGGGACTTGAGCG
SOX2 RNAi-1	GATCCGGACAGTTGCAAACGTGAA CTCGAGTTCACGTTTGCAACTGTC	AATTCAAAAAAGGACAGTTGCAA ACGTGAACTCGAGTTCACGTTTGC

	CTTTTTTG	AACTGTCCG
SOX2 RNAi-2	GATCCGGTTTGTAATATTTCTGTA CTCGAGTACAGAAATATTACAAAC CTTTTTTG	AATTCAAAAAAGGTTTGTAATATT TCTGTACTCGAGTACAGAAATATT ACAAACCG

Table S5. Antibodies used in this study.

Target	Application	Clone/Catalog #	Vendor
COPS3	WB, IF	ab79698	Abcam
COPS3	Co-IP, IHC	ab229807	Abcam
SOX2	Co-IP, ChIP	23064	Cell Signaling Technology
SOX2	WB, IF, IHC	11064-1-AP	Proteintech
CD133	Flow, IF, IHC	ab19898	Abcam
NANOG	WB, IF, IHC	14295-1-AP	Proteintech
Ubiquitin	WB	ab134953	Abcam
ITGB2	WB, IF, IHC	10554-1-AP	Proteintech
ITGB2	Co-IP	ab176540	Abcam
N-Cadherin	IF	22018-1-AP	Proteintech
E-Cadherin	IF	20874-1-AP	Proteintech
GAPDH	WB	60004-1-Ig	Proteintech
β-Actin	WB	66009-1-Ig	Proteintech
Rabbit IgG	Co-IP	30000-0-AP	Proteintech

Table S6. Primer Sequences Used for qPCR.

Primers names	Primers sequences (5’→3’)
COPS3-Forward	GACCTCAGGAGGCAGAGAAATA
COPS3-Reverse	GTTATGAAGCATGGCTGGGTTATTA
SOX2-Forward	GGAAAACCAAGACGCTCATG
SOX2-Reverse	ATCATGCTGTAGCTGCCGTTG
GAPDH-Forward	GGAAGCTTGTCATCAATGGAAATC
GAPDH-Reverse	TGATGACCCTTTTGGCTCCC

Table S7. Primer Sequences Used for ChIP-PCR.

Primers names	Primers sequences (5’→3’)
H3-Forward	5'-TGTGGCGCTCCGTGAAATTAG-3'
H3-Reverse	5'-CTGCAAAGCACCGATAGCTG-3'
NANOG-Forward	5'-AGGGTCTCACTTTGCAGCTC-3'
NANOG-Reverse	5'-TGGGCAACAGAATGAGACCC-3'
COPS3-Forward	5'-ATGAGGTTCACAGGGAAGGC-3'
COPS3-Reverse	5'-CTGAAGACCACAACAGCCCT-3'
CD133-Forward	5'-CGCTGCGATATGTTGGGTTG-3'
CD133-Reverse	5'-CTGGGGTCTGTCTGCTTCTG-3'
